# Fungal-assisted algal flocculation: application in wastewater treatment and biofuel production

**DOI:** 10.1186/s13068-015-0210-6

**Published:** 2015-02-15

**Authors:** Nazim Muradov, Mohamed Taha, Ana F Miranda, Digby Wrede, Krishna Kadali, Amit Gujar, Trevor Stevenson, Andrew S Ball, Aidyn Mouradov

**Affiliations:** Florida Solar Energy Centre, University of Central Florida, 1679 Clearlake Road, 32922 Cocoa, FL USA; School of Applied Sciences, Royal Melbourne Institute of Technology University, 3083 Bundoora, Melbourne, VIC Australia

**Keywords:** Microalgae, Fungi, Flocculation, Biofuel, Bioremediation, Pyrolysis, Renewable energy, Wastewater

## Abstract

**Background:**

The microalgal-based industries are facing a number of important challenges that in turn affect their economic viability. Arguably the most important of these are associated with the high costs of harvesting and dewatering of the microalgal cells, the costs and sustainability of nutrient supplies and costly methods for large scale oil extraction. Existing harvesting technologies, which can account for up to 50% of the total cost, are not economically feasible because of either requiring too much energy or the addition of chemicals. Fungal-assisted flocculation is currently receiving increased attention because of its high harvesting efficiency. Moreover, some of fungal and microalgal strains are well known for their ability to treat wastewater, generating biomass which represents a renewable and sustainable feedstock for bioenergy production.

**Results:**

We screened 33 fungal strains, isolated from compost, straws and soil for their lipid content and flocculation efficiencies against representatives of microalgae commercially used for biodiesel production, namely the heterotrophic freshwater microalgae *Chlorella protothecoides* and the marine microalgae *Tetraselmis suecica*. Lipid levels and composition were analyzed in fungal-algal pellets grown on media containing alternative carbon, nitrogen and phosphorus sources from wheat straw and swine wastewater, respectively. The biomass of fungal-algal pellets grown on swine wastewater was used as feedstock for the production of value-added chemicals, biogas, bio-solids and liquid petrochemicals through pyrolysis. Co-cultivation of microalgae and filamentous fungus increased total biomass production, lipid yield and wastewater bioremediation efficiency.

**Conclusion:**

Fungal-assisted microalgal flocculation shows significant potential for solving the major challenges facing the commercialization of microalgal biotechnology, namely (i) the efficient and cost-effective harvesting of freshwater and seawater algal strains; (ii) enhancement of total oil production and optimization of its composition; (iii) nutrient supply through recovering of the primary nutrients, nitrogen and phosphates and microelements from wastewater. The biomass generated was thermochemically converted into biogas, bio-solids and a range of liquid petrochemicals including straight-chain C12 to C21 alkanes which can be directly used as a glycerine-free component of biodiesel. Pyrolysis represents an efficient alternative strategy for biofuel production from species with tough cell walls such as fungi and fungal-algal pellets.

**Electronic supplementary material:**

The online version of this article (doi:10.1186/s13068-015-0210-6) contains supplementary material, which is available to authorized users.

## Background

In spite of substantial efforts worldwide to produce renewable biofuels, significant challenges still need to be overcome before microalgal-based biofuel production becomes cost effective and can impact the world’s supply of transport fuel [1-6]. The major challenges that need to be addressed before the development of a large-scale integrated algal industry include (i) optimization of microalgal harvesting/dewatering technologies; (ii) provision of a sustainable and renewable nutrient supply; (iii) improvement in oil content and composition; and (iv) increase in efficiency and reduction in the cost of lipid extraction.

Harvesting can account for up to 50% of the total cost of biodiesel production and is not economically viable for large-scale microalgal industry because of the significant energy requirements and/or the addition of costly chemicals (for reviews, see [7-15]). The main techniques used for harvesting microalgal cells include centrifugation, filtration, flocculation, gravity sedimentation and flotation [8,9,16-20]. Centrifugation can harvest about 90% of the microalgae; however, this comes with a high energy input cost, especially for a low value product such as biofuel [13]. Floatation is a method in which air or gas bubbles or flocculants attach to the microalgal cells carrying them to the surface [8,21]. Recently, Garg *et al*. (2014) showed that recovery of the marine microalgae *Tetraselmis* sp. can be increased up to 97.4% using improved froth flotation performance [22]. Filtration is efficient only for the large multicellular microalgae such as *Coelastrum proboscideum* and *Spirulina platensis*, and frequent filter replacement makes this method uneconomical [8,23]. This process is slow, although processing speed can be increased using the addition of flocculants [24]. Flocculation is the process by which algae forms clumps, pellets or pellet-like structures called flocs. Being negatively charged on the surface microalgal cells do not self flocculate, as the negative charge prevents aggregation under normal growth conditions [8,25-27]. Neutralizing and reducing the microalgal surface charge can be achieved by the application of chemical flocculants (inorganic and organic) and biological flocculants or by inducing an electrical impulse to neutralize the surface charge [16]. These methodologies are not universally successful and do not work for all microalgae strains [16,28].

The organisms that have been shown to induce efficient bioflocculation of microalgae are bacteria and fungi [13,29-31]. The gram-positive bacteria *Solibacillus silvestris* and *Bacillus* sp. both showed a flocculation efficiency of up to 90% with the marine microalgae *Nannochloropsis oceanica* [30]. An efficient bioflocculant has been isolated from the autoflocculating *Scenedesmus* and *Chlorella vulgaris* (*C. vulgaris*) microalgae when they were grown in wastewater [29]. Fungal self-pelletization has been observed for numerous filamentous strains and can be explained by coagulative and non-coagulative mechanisms [17,32-35]. The coagulative mechanism involves spore coagulation leading to the developments of aggregates/pellets. Representatives of *Aspergillus* sp., *Basidiomycete* sp. and *Phanerochaete* sp*.* produce dense spherical aggregates through coagulative mechanism [17,33]. The non-coagulative mechanism suggests that the spores germinate into hyphae, which then will intertwine into pellets. Representatives of *Rhizopus* sp., *Mucor* sp. and *Penicillium* sp. display the non-coagulative mechanism [17,33]. Fungal-assisted microalgal harvesting technology does not require the addition of chemicals or inputs of energy, and a number of microalgal strains have been shown to be efficient [17,33,36-40]. If this technology can be applied to commercially important freshwater and seawater algal species, it can offer a solution to one of the major problems associated with the energy-intensive and costly harvesting processes.

The detailed mechanisms of the fungal-algal interactions are still not clear. It was suggested that the algae have a negative surface charge (−23.7 mV) due to the presence of proton-active carboxylic, phosphoric, phosphodiester, hydroxyl and amine functional groups [17,23]. Fungal hyphae and mycelia contain polysaccharides that have been shown be positively charged (+46.1 mV) and therefore can potentially neutralize the negative charges on the algal surface, enabling attachment to the fungal cell wall.

Natural symbiosis between fungi, microalgae and cyanobacteria, known as lichens, has existed since plants evolved from green algae, more than 400 million years ago; and these lichens are covering 6% of Earth’s land surface [41] (Additional file 1). In this mutually beneficial symbiosis, fungi consume the sugars and nutrients produced by the algae through photosynthesis; in return, the fungus affords protection to the algae by retaining water, serving as a larger capture area for mineral nutrients and, in some cases, provides minerals obtained from the substrate [42]. This suggests that fungal-microalgal pellets can also function as a self-sufficient system which can potentially improve the overall economics of a large-scale integrated microalgal industry.

Lipid production by oleaginous microorganisms is a promising route to produce crude oil material for the production of biodiesel. Microalgal strains have been widely used by researches and biotechnology companies because they are able to accumulate large amounts of neutral lipids (up to 60% of their dry weight). The profiles of their transesterification (TE) products revealed a high content of fatty acids, similar to conventional vegetable oils used for biodiesel production. The application of oleaginous fungi for biodiesel production is, to date, limited in spite of obvious advantages over conventional plant and microalgal resources. Oleaginous fungi can accumulate >20% (w/w) of their dry cell mass in the form of neutral lipids, with a high content of saturated and monounsaturated fatty acids such as palmitic (C16:0), stearic (C18:0) and oleic (C18:1) acids commonly used for biodiesel production. Fungi can be easily grown in bioreactors with rapid growth rates unaffected by light intensity and duration (photoperiod) and are able to utilize a wide range of lignocellulosic waste biomass as renewable carbon sources and wastewater nutrients as sources of nitrogen (N) and phosphorus (P) [9,43,44]. Moreover, pelletization of fungal cells during growth in liquid media makes their harvest much easier and cheaper than the isolation of the microalgal strains (for review, see [17]).

Unlike plant and most of microalgal cells, fungal cells contain tough cell walls with a complex structure composed of extensively cross-linked chitin, glucans and other polymers [17,45]. Some microalgal strains, such as *Nannochloropsis occulata*, also have very tough cell walls which require special pretreatment prior to extraction of intracellular lipid [46]. This makes the extraction procedure from these species challenging.

Pyrolysis has recently attracted increased attention due to a number of advantages, including relatively mild operational conditions and the resultant production of several valuable products: pyrolysis gas, bio-oil and bio-solids (bio-char and mineral ash). In most cases, bio-oil is the main target product of pyrolysis because it can be further processed via catalytic hydrodeoxygenation (CHDO) and/or hydrocracking to liquid hydrocarbon products similar to petroleum-derived fuels. So far, most of the pyrolysis research has concentrated on lignocellulosic materials such as wood, straws and stalks [47,48]. The thermal conversion of algae into bio-oil has also been intensively studied over recent years because of their ability to produce substantial biomass with high oil content [49-52]. Recently, we have reported pioneering studies on the pyrolysis of aquatic plants and microalgal representatives, which showed great potential as feedstock for the production of bio-oil and bio-char [39,53-56]. Some of these species were used for the efficient bioremediation of animal and mining wastewaters and represent an attractive, ecologically friendly and potentially cost-effective solution for the conversion of waste biomass into sustainable bioenergy [39,53,55]. In spite of the impressive biomass production rate, the high content of carbohydrates, proteins and lipid level and fatty acid composition, to our knowledge, there is no report on pyrolysis of oleaginous fungi.

In this work, we screened 33 fungal strains isolated from compost, straws and soil, a rich source of fungi for lipid concentrations and flocculation efficiencies. For the first time: (i) flocculation efficiency was tested against two commercially important microalgal strains, the heterotrophic freshwater algae, *Chlorella protothecoides* (*C. protothecoides*), and the marine species, *Tetraselmis suecica* (*T. suecica*), widely used for biodiesel production [57-61]; (ii) lipid level and fatty acid composition were analyzed in fungal-algal pellets grown on alternative carbon sources; (iii) fungal-algal pellets grown on swine wastewater were used as feedstock for production of value-added products, biogas, bio-solids and liquid petrochemicals through pyrolysis. Our research showed that co-pelletization of fungal and microalgal cells can not only increase the total lipid production but also tailor its composition, via combinations of different microalgal and fungal strains. Co-cultivation of algae and filamentous fungal cells also increased the recovery of N/P nutrients from wastewater.

## Results and discussion

### Phenotypic and biochemical aspects of fungal-assisted algae flocculation

#### Screening for oleaginous fungal strains

A total of 33 fungal strains, isolated from compost, straws and soil, were screened for intracellular oil accumulation (Additional files 2 and 3). Screening was designed on the assumption that oil accumulated in fungal cells grown on enrichment broth (EB) containing 100 g/L of glucose will be utilized for growth on carbon-free media (CFM). As a result, strains with higher levels of accumulated oil will grow faster on CFM plates [62]. Additional file 3 compares the growth rates of analyzed fungal strains after 12 and 24 h of growth on CFM. Based on growth rates, 15 strains were selected for lipid extraction/quantification and algal flocculation rate analysis.

**Figure 1:**
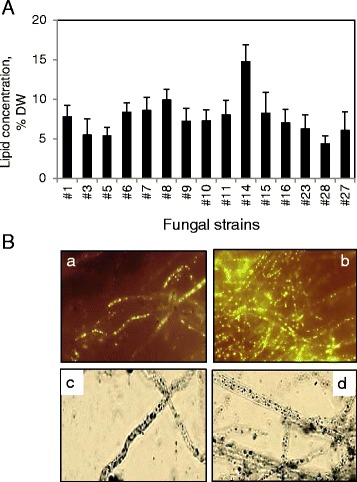
**Lipid production in fungal isolates. (A)** The lipid concentrations in cultured fungal strains; **(B)** microscopic analysis of oil bodies accumulation in *A. fumigatus* (a, c) and *M. circinelloides* (b, d) using Nile red (a, b) and Sudan black (c, d).

Concentration of lipids in the selected 15 fungal strains after growth in potato dextrose broth (PDB) containing 20 g/L of glucose were found to be between 4.3% and 14.7% of dry weight (DW). Two strains, #8 [*Aspergillus fumigatus* (*A. fumigatus*)] and #14 [*Mucor circinelloides* (*M. circinelloides*)], showed the highest lipid content with levels of 9.9% and 14.7% DW, respectively (Figure 1A). *M. circinelloides* strains have previously been shown to have an oil content up to 23% DW [35,44]; however, the strain used in this study showed a modest oil content, close to the lipid concentration found in *M. circinelloides* strain by Xia *et al*. (2011 and 2014) [35,44]. The *A. fumigatus* and *M. circinelloides* strains showed visible oil bodies when stained with Nile red and Sudan black (Figure 1B). The 15 selected fungal strains showed different rates of self-pelletization, producing loose and dense spherical aggregates (Additional file 4A). Pellet sizes could be changed by altering the speed of rotation used for growth in liquid broth (Additional file 4B).

#### Flocculation efficiency of microalgal cells by fungal cells

**Figure 2:**
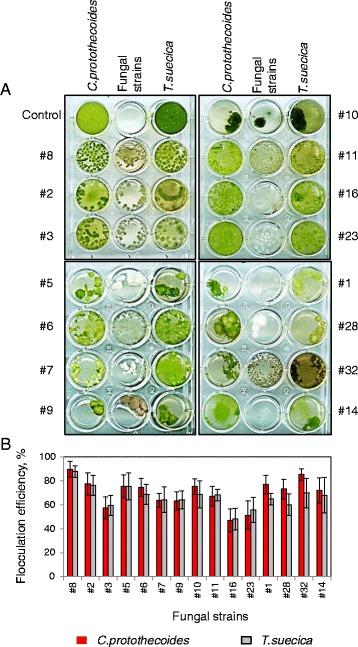
**Flocculation of** ***C. protothecoides*** **and** ***T. suecica*** **by 15 fungal strains. (A)** 12-well microtitre plate experiment: fungal pellets were mixed with suspensions of *C. protothecoides* (left wells) and *T. suecica* (right wells) for 24 h. Controls: microalgal suspensions grown without fungi (top wells); fungal cultures grown alone (middle wells). **(B)** Flocculation efficiency measured by reduction in optical densities, cell numbers and chlorophyll concentrations of uncaptured algal cells after 24 h of co-cultivation.

As a first screen for microalgal flocculation efficiency, pelletized fungal cultures were mixed with medium cell density cultures of *C. protothecoides* (grown mixotrophically, 1.5 × 10^7^ cells/mL) and *T. suecica* (grown autotrophically, 6.5 × 10^6^ cells/mL) in 12-well microtitre plates (Figure 2A). Efficiency of microalgal harvesting was measured by reduction in optical densities, cell numbers and chlorophyll concentrations of uncaptured algal cells after 24 h of co-cultivation with fungal pellets (Figure 2B). Based on the high flocculation efficiency of both microalgal strains, along with the production of dense spherical pellets, *A. fumigatus* (#8) was selected for a further round of flocculation tests. Efficient flocculation of the other microalgal representative have previously been shown for a number of filamentous fungal strains, including representatives of *Aspergilium* sp., *A. fumigatus, A. niger*, *A. oryze* and *A. nomius* [32,33,36-40].

**Figure 3:**
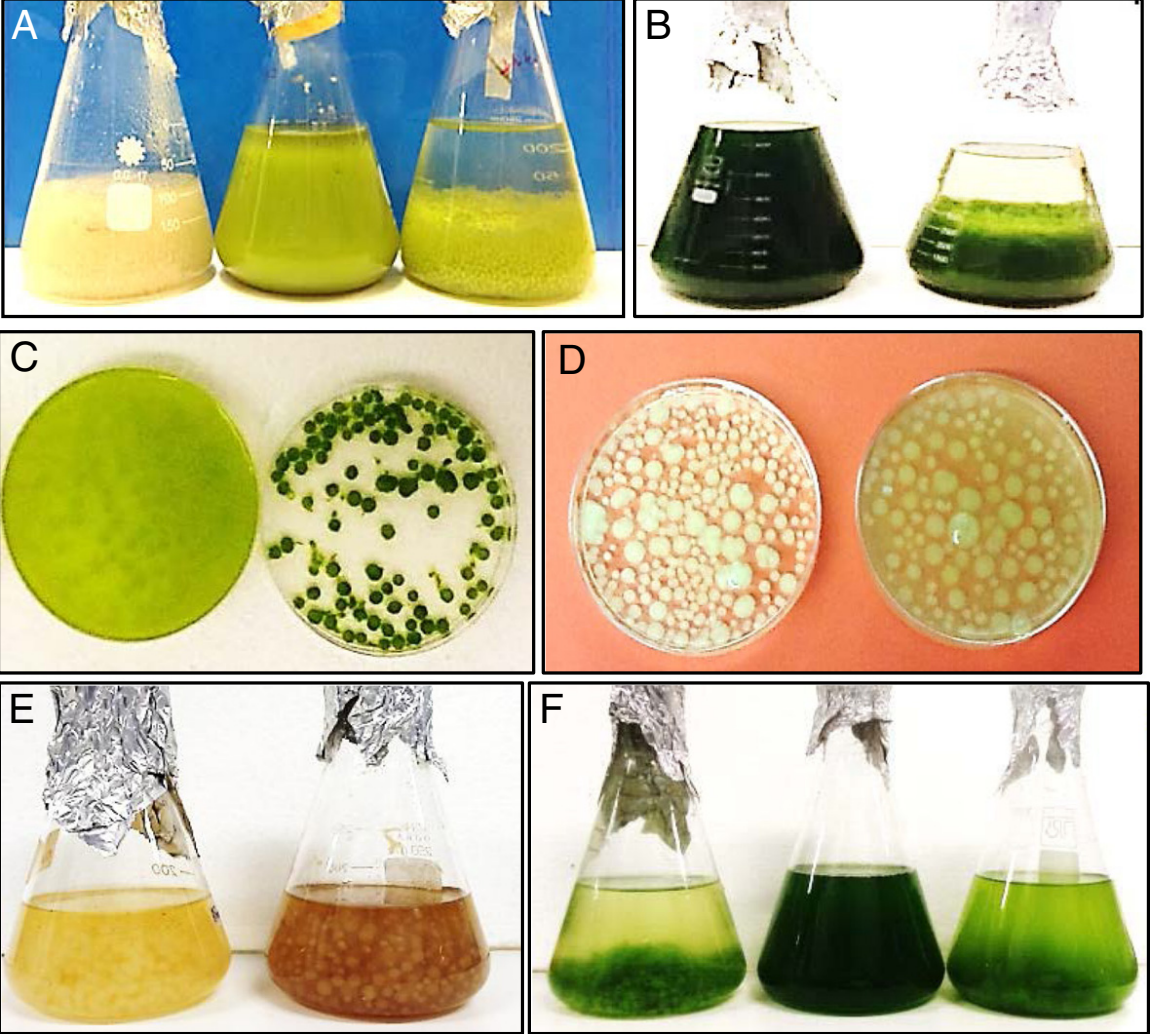
**Flocculation of microalgal strains by** ***A. fumigatus*** **.** **(A)** Flocculation of *C. protothecoides*: *A. fumigatus* culture (left); mixotrophically grown *C. protothecoides* culture (middle); *A. fumigatus/C. protothecoides* pellets (right). **(B)** Flocculation of *T. suecica*: autotrophically grown *T. suecica* culture (left); *A. fumigatus/T. suecica* pellets (right). **(C)** *T. suecica* culture mixed with *A. fumigatus* pellets, time = 0 (left); 24 h later (right). **(D, E)** *A. fumugatus* pellets grown PDB (left) and 1% TWS (right). **(F)** Flocculation of *T. suecica*: *A. fumigatus/*PDB*-T. suecica* pellets (left); original *T. suecica* culture (middle); *A. fumigatus/*TWS-*T. suecica* pellets (right).

**Figure 4:**
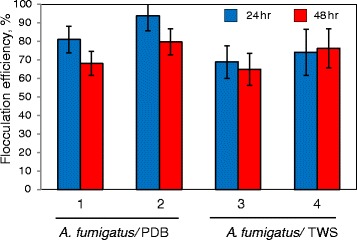
**Flocculation efficiency of algal strains by** ***A. fumigatus.*** Flocculation of *C. protothecoides* by *A. fumigatus/*PDB (1) and *A. fumigatus/* TWS pellets (3); flocculation of *T. suecica* by *A. fumigatus/*PDB pellets (2) and *A. fumigatus/* TWS pellets (4).

For the larger scale flocculation experiments, *A. fumigatus* pellets produced on PDB (*A. fumigatus*/PDB) were mixed with high cell density cultures of *C. protothecoides* (1 to 3 × 10^9^ cells/mL grown mixotrophically) and *T. suecica* (7 to 12 × 10^8^ cells/mL, grown autotrophically). *A. fumigatus* showed up to 90% flocculation after the first 24 h of co-cultivation with no obvious differences in flocculation efficiency between freshwater and seawater microalgal species (Figures 3 and 4). The concentrations of uncaptured microalgal cells were slightly increased after 24 h (shown by a decrease in flocculation efficiency), which can be explained by their release from the fungal filaments and/or by independent growth of uncaptured microalgal cells in the media [17,32,36,39]. Additional file 5 shows that within fungal-algal pellets, microalgal cells do not just get trapped within fungal filaments but get attached to them. Examination of *T. suecica* cells showed the absence of visible algal cell walls after co-culturing with fungal strains. This can be explained by the secretion of hydrolytic enzymes by fungal cells in the presence of the microalgal cells (Additional file 5).

To test the efficiency of microalgal flocculation by *A. fumigatus* grown on alternative carbon sources, fungal spores were grown on carbon-free broth containing 1% acid-treated wheat straw [TWS (*A. fumigatus*/TWS)]. *A. fumigatus*/TWS pellets showed no differences in size compared to *A. fumigatus* pellets grown on PDB media *(A. fumigatus*/PDB) (Figure 3). In order to assess the potential anti-algal effect of chemicals produced after digestion of TWS by *A. fumigatus*, microalgal cells were grown in the presence of 5% and 20% of media collected 72 h after incubation of *A. fumigatus* with TWS. Additional file 6 shows that 20% of added media led to the severe suppression of microalgal growth. To avoid toxic effect, *A. fumigatus*/TWS pellets were washed before mixing with microalgal cultures. The *A. fumigatus*/TWS pellets showed slightly lower flocculation rates than *A. fumigatus*/PDB pellets (Figure 4). This can be explained by (i) the effect of residual amount of toxic chemicals in the growth media [63] and (ii) microalgal cell wall digestion by the cocktails of hydrolytic enzymes secreted from *A. fumigatus* in the presence of TWS. No significant increase in the numbers of uncaptured microalgal cells was detected in the media after 24 h of co-cultivation.

#### Lipid production in fungal-microalgal pellets

Mono-cultured *A. fumigatus*/PDB pellets before mixing with microalgal cultures showed lipid content of 10% of its DW biomass and a lipid yield of 71 mg/L (time 0, Table 1). Not surprisingly, *A. fumigatus*/TWS pellets showed significantly lower lipid content, 2.8% of DW with a lipid yield of 16 mg/L (time 0). Lipid concentration in mono-cultured *T. suecica* (7 to 12 × 10^8^ cells/mL) before mixing with *A. fumigatus* pellets was 15% of its DW with a lipid yield of 86 mg/L (time 0). Mono-cultured *C. protothecoides* (1 to 3 × 10^9^ cells/mL) grown under mixotrophic conditions showed a lipid content of 26% of its DW and a lipid yield of 172 mg/L (time 0). For flocculation assessment experiments, fungal pellets were mixed with *C. protothecoides* or *T. suecica* cultures and the mixtures were then shaken at 150 rpm for 48 h.

**Table 1:** **Biomass and lipid concentrations in** ***A. fumigatus*** **and microalgal strains grown in mono-cultures and co-cultures**

	**Fungi/microalgae monocultures, 0 h**			**Fungi/microalgae monocultures, 48 h**			**Microalgae +** ***A. fumigatus/TWS*** **,** **co-cultures, 48 hr**			**Microalgae +** ***A. fumigatus/PDB*** **,** **co-cultures, 48 hr**		
*Species*	Biomass	Lipid	Lipid yield	Biomass	Lipid	Lipid yield	Biomass	Lipid	Lipid yield	Biomass	Lipid	Lipid yield
	(g/L)	(%)	(mg/L)	(g/L)	(%)	(mg/L)	(g/L)	(%)	(mg/L)	(g/L)	(%)	(mg/L)
*Fungi*												
*A. fumigatus/TWS*	0.66 ± 0.1	2.80 ± 0.3	16.68 ± 4.3	1.11 ± 0.2	3.36 ± 0.4	37.71 ± 8.4	NA	NA	NA	NA	NA	NA
*A. fumigatus/PDB*	0.71 ± 0.1	10.10 ± 3.2	71.09 ± 15.3	2.21 ± 0.5	11.50 ± 3.3	240.20 ± 41.9	NA	NA	NA	NA	NA	NA
*Freshwater microalgae*												
*C. protothecoides*	0.66 ± 0.1	26.20 ± 4.1	172.23 ± 48.	2.25 ± 0.4	28.20 ± 6.4	699.70 ± 120.4	6.61 ± 1.1	12.35 ± 4.4	755.34 ± 122.0	8.96 ± 2.1	21.35 ± 4.5	2041.96 ± 440.6
*Marine microalgae*												
*T. suecica*	0.65 ± 0.1	15.10 ± 3.8	86.54 ± 20.0	1.77 ± 0.4	13.70 ± 2.5	215.55 ± 50.6	4.40 ± 1.1	6.1 ± 1.7	268.84 ± 53.2	4.49 ± 0.9	12.10 ± 3.1	578.29 ± 210.7

Lipid production in the fungal-microalgal pellets showed complex profiles reflecting at least three main factors: biomass production and lipid concentrations in fungal and microalgal cells before and during co-cultivation and the efficiencies of bioflocculation. After 48 h of co-culture of *A. fumigatus*/PDB with oleaginous *C. protothecoides*, lipid concentration in pellets was found lower than in mono-cultured microalgae but higher than in mono-cultured *A. fumigatus,* suggesting that the microalgal strain was the main contributors to the total content of lipids. The total lipid yields (mg/L) in these pellets were found to be higher than the additive lipid content of mono-cultured *C. protothecoides* and *A. fumigatus* (Table 1). In *A. fumigatus*/PDB-*T. suecica* pellets, lipid concentration and yield were not very different from mono-cultured *T. suecica* and *A. fumigatus.* These lipid yields correlated with generated biomasses of the *A. fumigatus*/PDB-microalgal pellets. Lipid concentrations of *A. fumigatus*/TWS pellets with *C. protothecoides* and *T. suecica* were found to be lower than in mono-cultured microalgae and significantly higher than in mono-cultured *A. fumigatus/*TWS suggesting that microalgae were essential contributors to the total level of lipids in these algal-fungal pellets. Lipid yields (mg/L) in all pellets were also found to be higher than in mono-cultured algal and fungal strains and correlated with the amount of generated biomass.

The synergistic effects of co-cultivation of *A. niger*, *A. fumigatus*, *A. oryzae* and *C. echinulata*, with microalgal representatives on biomass production and lipid yields, have also been reported by [32,36,38,39]. Enhancement of fungal biomass can be explained by utilization of the microalgal cell wall carbohydrates as carbon source by fungal cells. Cellulase activity was shown to be induced upon co-cultivation of *C. echinulata* with *C. vulgaris* [36]. This activity was absent in the mono-cultured *C. echinulata*. The composition of microalgal cell walls vary between different strains; however, cellulose is reported as the main structural component of the cell wall for some [64-66]. Treatment with external cellulase resulted in a significant increase in sugar concentration in the culture of *C. vulgaris* [65-67]. In our experiments, the potential *A. fumigatus* cellulase activity was correlated with the observation of cell wall-free microalgal protoplasts found either attached to the fungal cells or remaining uncaptured in cultivation media. The saprophytic behavior of the fungal component of natural lichens secreting phenol oxidases, peroxidases and cellulases benefits their growth when algal photosynthesis is limited [68].

#### Fatty acid composition of fungal-microalgal pellets

**Figure 5:**
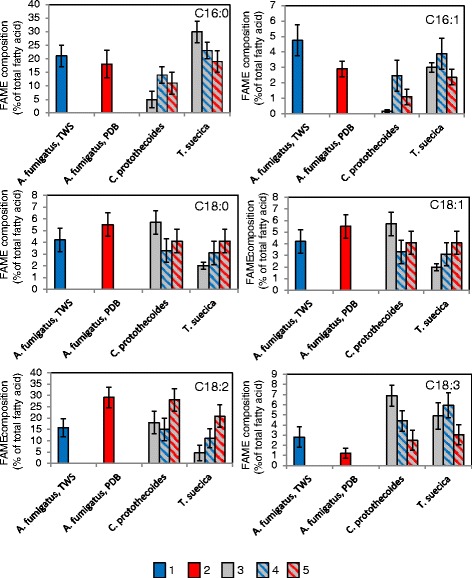
**Fatty acid composition of** ***A. fumigatus, C. protothecoides, T. suecica*** **and fungal-algal pellets.** 1) *A. fumigatus*/TWS pellets; 2) *A. fumigatus*/PDB pellets; 3) algal strains; 4) *A. fumigatus*/TWS-algal pellets; 5) *A. fumigatus*/PDB-algal pellets.

Fatty acids in oleaginous fungi are represented mainly by palmitate, C16:0, stearate, C18:0, oleate, C18:1, and linoleate, C18:2 [35,44,62,69-71]. Fatty acid composition (measured by composition of fatty acid methyl esters (FAMEs)) of *A. fumigatus*/PDB and *A. fumigatus*/TWS pellets with *C. protothecoides* and *T. suecica* is shown in Figure 5. Fatty acid composition of *A. fumigatus/*PDB is dominated by palmitate, C16:0 (ca 18%), and linoleate, C18:2 (ca 30%) (Figure 5). *A. fumigatus*/TWS pellets showed higher concentration of palmitoleate, 16:1, and linolenate, 18:3, and lower concentration of linoleate, C18:2, than *A. fumigatus/*PDB, *C. protothecoides* and *T. suecica* cells which showed almost opposite profiles of fatty acid compositions: *T. suecica* was significantly higher in palmitate, C16:0, and palmitoleate, C16:1, while *C. protothecoides* contained a higher proportion of stearate, oleate and linoleate (C18:0, C18:1 and C18:2, respectively). Similar compositions of FAMEs in *C. protothecoides* and *T. suecica* have been reported [51,61,72]. Fatty acid composition of the fungal-algal pellets reflected the levels and compositions in both fungal and algal strains and the efficiencies of their co-pelletization. Microalgae were the main contributors of the linolenate, C18:3. *A. fumigatus* was also a main contributor of C16:0 and C16:1 for *C. protothecoides* and C18:0 and C18:2 for *T. suecica*. Co-contributions of fatty acids from fungal strains and microalgal strains were previously shown [32,33,36,39] .

#### Swine wastewater as an alternative source of nutrients for fungal-microalgal pellets

**Figure 6:**
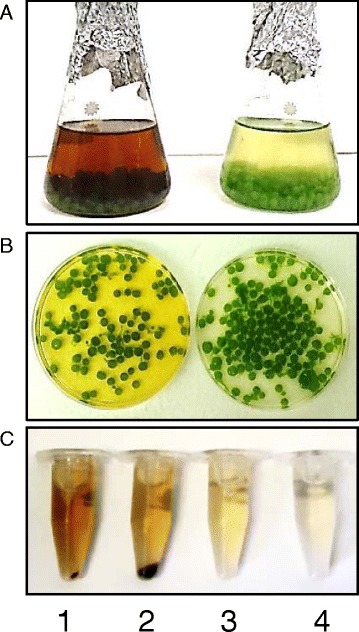
**Application of** ***A. fumigatus*** **/** ***C. protothecoides*** **pellets for swine wastewater treatment. (A, B)** *A. fumigatus/C. protothecoides* pellets with 25% wastewater: t = 0 (left); 48 h later (right); **(C)** samples of 25% wastewater before (1) and after treatment with *C. protothecoides* (2), *A. fumigatus* (3) and *A. fumigatus/C. protothecoides* (4).

**Figure 7:**
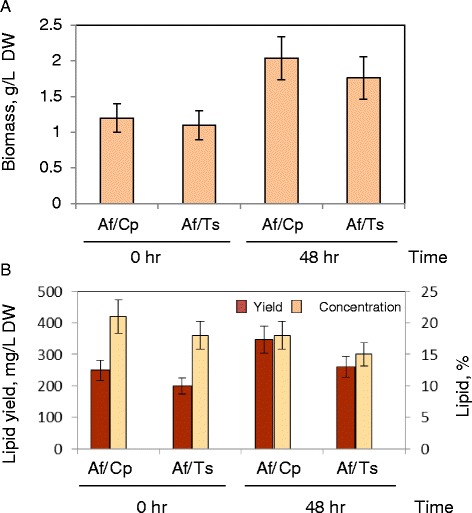
**Biomass and lipid production.** Biomass and lipid production of *A. fumigatus/C. protothecoides* and *A. fumigatus/T. suecica* pellets grown in 25% swine wastewater. Af/Cp: *A. fumigatus*/*C. protothecoides* pellets; Af/Ts: *A. fumigatus*/*T. suecica* pellets.

Both microalgal and fungal cells have been extensively used as efficient bio-remediating agents for wastewater treatment [7,9,10,25,43,73-76]. We assessed the ability of *A. fumigatus/C. protothecoides* (Af/Cp) and *A. fumigatus/T. suecica* pellets (Af/Ts) to grow and uptake nutrients (NH_4_^+^-N and PO_4_^−3^-P) from diluted anaerobically digested swine manure wastewater (ASW) prepared from swine lagoon wastewaters (Table 2, Figure 6). For these experiments, the swine wastewater was diluted to 10% and 25% with sterile tap water for Af/Cp and seawater for Af/Ts. After 48 h of Af/Cp incubation in 25%, wastewater concentrations of NH_4_^+^-N was reduced from 164.3 to 43.2 mg/L (73.9% uptake) and the concentration of PO_4_^−3^-P was reduced from 38.7 to 17.2 mg/L (55.6% uptake). This removal efficiency was higher than the efficiency of NH_4_^+^-N, and PO_4_^−3^-P removal was achieved separately by *C. protothecoides* (36% and 25%, respectively) and by *A. fumigatus* (46% and 20%, respectively, Table 2). In 10% ASW, both nutrients were practically removed after 48 h of incubation with Af/Cp (93% removal for NH_4_^+^-N and 87% removal of PO_4_^−3^-P). Growing in different dilutions of ASW in seawater, *A. fumigatus* showed no obvious differences in nutritional uptake compared to ASW diluted with tap water. In spite of the fact that *T. suecica* alone showed lower rates of uptake of NH_4_^+^-N and PO_4_^−3^-P than *C. protothecoides*, incubation of Af/Ts pellets in 10% and 25% ASW diluted in seawater showed similar uptake rates to Af/Cp. Efficient wastewater treatment by *Aspergillus* sp*./C. vulgaris* pellets was shown by Zhou *et al*. (2012, 2013) [37,38]. Wastewater with much lower concentrations of NH_4_^+^ and similar concentration of PO_4_^3+^ (51.2 mg/L for both) was used in this work. Nutrient uptake by Af/Cp and Af/Tc pellets led to 1.7- and 1.6-fold increase in their biomass production after 48 h of treatment (Figure 7). The lipid yield was increased by 1.3-fold (for both pellets), which associated with slightly reduced lipid concentrations of 18% and 15% DW for Af/Cp and Af/Tc, respectively.

**Table 2:** **Concentrations of nutrients in diluted swine wastewater before and after treatment with** ***C. protothecoides, T. suecica*** **and their pellets with** ***A. fumigatus***

**Concentrations of nutrients diluted in tap water and sea water**								
*Composition*	*ASW*		*C. protothecoides*		*A. fumigatus*		*C. protothecoides + A. fumigatus*	
*Dilutions in tap water*	*NH*_*4*_ *-N, mg/L*	*PO*_*4*_ *−P, mg/L*	*NH*_*4*_ *-N, mg/L*	*PO*_*4*_ *−P, mg/L*	*NH*_*4*_ *-N, mg/L*	*PO*_*4*_ *−P, mg/L*	*NH*_*4*_ *-N, mg/L*	*PO*_*4*_ *−P, mg/L*
*ASW, 100%*	680.7 ± 23.1	145.4 ± 13.7	NA	NA	NA	NA	NA	NA
*ASW, 25%*	164.3 ± 13.2	38.7 ± 3.4	104.8 ± 12.1	25.0 ± 5.1	98.8 ± 12.9	19.0 ± 6.1	43.2 ± 11.9	17.2 ± 3.1
*ASW, 10%*	66.1 ± 4.3	16.1 ± 3.0	29.9 ± 6.2	8.7 ± 2.6	18.9 ± 4.4	6.7 ± 2.8	4.4 ± 4.6	3.1 ± 1.7
*Composition*	*ASW*		*T. suecica*		*A. fumigatus*		*T. suecica + A. fumigatus*	
*Dilutions in seawater*	*NH*_*4*_ *-N, mg/L*	*PO*_*4*_ *−P, mg/L*	*NH*_*4*_ *-N, mg/L*	*PO*_*4*_ *−P, mg/L*	*NH*_*4*_ *-N, mg/L*	*PO*_*4*_ *−P, mg/L*	*NH*_*4*_ *-N, mg/L*	*PO*_*4*_ *-P, mg/L*
*ASW, 100%*	699.4 ± 25.1	169.2 ± 18.1	NA	NA	NA	NA	NA	NA
*ASW, 25%*	168.8 ± 17.0	45.0 ± 4.0	124.1 ± 10.7	31.0 ± 5.1	99.3 ± 11.1	25.8 ± 8.2	63.9 ± 17.2	19.0 ± 5.6
*ASW, 10%*	67.9 ± 5.5	18.7 ± 3.2	41.3 ± 4.2	10.2 ± 3.1	20.3 ± 5.4	8.2 ± 1.9	3.9 ± 5.4	4.2 ± 2.1

### Pyrolysis of *C. protothecoides, A. fumigatus* and *A. fumigatus/C. protothecoides* pellets

#### *Thermogravimetric analysis of* C. protothecoides*,* A. fumigatus *and* A. fumigatus/C. protothecoides *pellets*

The biomass of *C. protothecoides, A. fumigatus* and *A. fumigatus/C. protothecoides* pellets collected after bioremediation of wastewater were subjected to thermogravimetric (TGA) and derivative thermogravimetric (DTG) analyses in order to determine their thermal stability/behavior pattern over the range of temperatures of 25°C to 950°C. TGA/DTG analysis provides a means for conducting proximate analysis of the samples as well as the preliminary mechanistic studies of the biomass pyrolysis process. Proximate analysis of the dry algal biomass samples, *C. protothecoides, A. fumigatus* and *A. fumigatus/C. protothecoides*, is shown in Table 3. It was observed that the volatile matter (both primary and secondary) content of all samples tested was similar; however, the amount of fixed carbon and ash was found to be lower in the fungal sample.

**Table 3:** **Proximate analysis of the** ***C. protothecoides, A. fumigatus/C. protothecoides*** **and** ***A. fumigatus*** **samples determined by TGA method**

**Sample**	**Moisture (25°C to 120°C), %**	**Primary volatiles (120°C to 650°C), %**	**Secondary volatiles (650°C to 950°C), %**	**Total volatiles, (120°C to 950°C), %**	**Fixed carbon, %**	**Ash, %**
*C. protothecoides*	4.7	67.1	4	71.1	20.2	4.1
*A. fumigatus/C. protothecoides*	6	67.6	4.4	72	18.5	3.5
*A. fumigatus*	9.2	67.4	5.4	72.8	15.5	2.5

**Figure 8:**
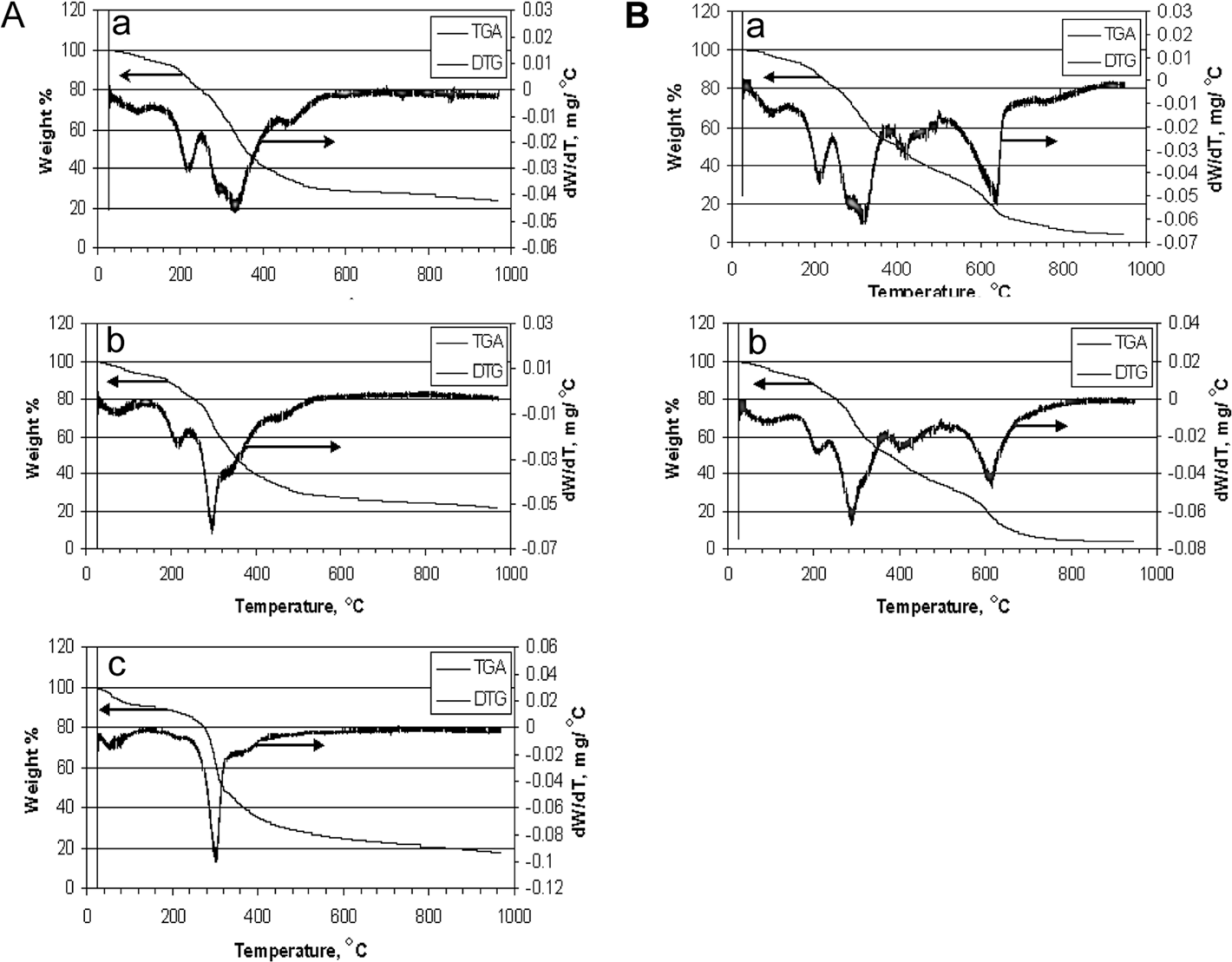
**Thermogravimetric analysis of biomasses. (A)**. TGA/DTG analysis of algae biomass samples in a He atmosphere. Heating rate: 20°C/min. a) *C. protothecoides*, b) *A. fumigatus*/*C. protothecoides*; c) *A. fumigatus.* **(B)** TG/DTG analysis of algae biomass samples in air atmosphere. Heating rate: 20°C/min. a) *C. protothecoides*, b) *A. fumigatus*/*C. protothecoides*.

Figure 8A shows the results of TGA/DTG analyses of all three samples in inert He atmosphere within the temperature range of 25°C to 950°C and a heating rate of 20°C/min. The TGA profiles of all samples show a significant weight loss in the temperature range of 200°C to 400°C. This was followed by an intermediate region (400°C to 600°C) and the rather slow rate of weight loss at temperatures above 600°C. The DTG curves of the tested samples in He atmosphere show certain similarities and also some distinct differences. Peaks in the low temperature region at about 60°C and 200°C can be attributed to dehydration processes involving water molecules adsorbed on the sample surfaces and those bound within the inner cells of the algae samples, respectively. It is noteworthy that *A. fumigatus* shows a very weak peak at 200°C. The majority of volatile compounds from all samples are released in the temperature range of 240°C to 400°C where major thermal degradation processes occur. During this stage, bio-polymers are thermally decomposed with less stable molecules breaking apart first. All three samples showed a weak peak at about 450°C. A comparison of TGA/DTG profiles of algal samples with that of terrestrial biomass such as poplar [77] saw dust [78] shows dissimilarities in that the intensive peak in algal DTG profile was shifted by about 50°C to a lower temperature range compared to the intensive peak in plant biomass DTG.

The results of TGA/DTG analysis are consistent with the complex structure of algal biomass, comprising several classes of natural compounds (proteins, lipids, carbohydrates), characterized by a distinct thermal stability signature. The peaks in the low-temperature range of 200°C to 220°C (medium intensity for *C. protothecoides* and *A. fumigatus/C. protothecoides* and weak for and *A. fumigatus*) can be attributed to decomposition and evaporation of oily compounds and other thermally unstable functional groups of various natural compounds. Similar observations were reported by other authors who pointed to degradation of oily compounds within the temperature range of 170°C to 230°C [79]. The intensive double peak in the DTG curve of *C. protothecoides* and *A. fumigatus/C. protothecoides* at 280°C to 350°C is in the same temperature range as the degradation temperature of plant hemicellulose (e.g. [77,78]. A cellulose peak typically manifests itself at the temperature range of 350°C to 370°C [77,78]. The reported results of DTG analysis of macroalgal species confirmed that hemicellulose degrades faster than cellulose with the latter breaking in the 250°C to 407°C temperature range [78]. Based on the above data, the double peak in DTG of *C. protothecoides* could be attributed to the hemicellulose-cellulose pair. In the DTG profile of *A. fumigatus/C. protothecoides*, the higher temperature peak (approximately 350°C) was more intense than that in the DTG of *C. protothecoides*, which may imply that the cellulose content of these samples was lower compared to mono-cultured algae.

The presence of raw protein in the tested samples could be obscured by an intensive hemicellulose peak. It was reported that the DTG curve of marine microalgae exhibited an intensive peak at 285°C (at a heating rate of 20°C/min), which was assigned to protein [80]. The relatively low thermal stability of proteins was also emphasized by other authors; for example, according to [81], the thermal stability of proteins is limited to about 200°C. The weak peak at about 460°C in DTG of *C. protothecoides* and *A. fumigatus/C. protothecoides* occurs where thermal degradation of thermally stable bio-polymers, such as lignin, typically takes place [77,82]. The thermal degradation of lignin in macroalgae [83] and plants [77,82] was also reported to occur at about the same temperature range. The occurrence of this peak may indicate the presence of lignin-like compounds in *C. protothecoides* and *A. fumigatus/C. protothecoides* (in DTG of *A. fumigatus*, this peak is very weak but still visible).

Figure 8B depicts the results of TGA/DTG analyses in an oxidative atmosphere (air) in the same temperature range of 25°C to 950°C and a heating rate of 20°C/min. The DTG profile of *A. fumigatus* is fairly similar to that of *A. fumigatus/C. protothecoides* (not shown). It is evident that the DTG profiles of thermal degradation of the samples in air were markedly dissimilar to those obtained in an inert (He) atmosphere. In particular, there are two distinct areas of a significant weight loss in the oxidizing atmosphere, as opposed to one area in inert atmosphere; these two areas are separated by the temperature gap of more than 300°C. The low-temperature peak at about 200°C and an intermediate-temperature peak at 280°C to 350°C are present in the DTG profiles in He. These peaks remain almost unchanged in the corresponding DTG profiles in air. This observation indicates that the oxidizing atmosphere only slightly affects the thermal degradation of the samples in a low-to-intermediate temperature range (that is, up to 400°C). The relatively weak peak at 420°C can be assigned to the oxidative thermal degradation of lignin-like compounds. The intensive peaks in the high temperature region of 620°C to 640°C (which are absent in the DTG curves in He) result from the combustion of bio-chars. A similar two-peak pattern were previously reported by [78,84] with regard to TGA/DTG analysis of straw, waste wood and duckweed, respectively, although in these cases the high temperature peak was observed at a somewhat lower range of temperatures of 460°C, 440°C, and 560°C, respectively (at the same heating rate of 20°C/min).

Mechanistically, the above experimental observations can be explained in terms of two concurrent pathways: (a) initial pyrolysis of biomass to volatile matter and char followed by their combustion and (b) direct combustion of biomass to oxidation products (CO_2_, H_2_O). The presence of the same peaks in the low- and intermediate-temperature range in the DTG profiles of biomass samples in both inert and oxidizing atmosphere points to the prevalence of pathway a compared to b. A similar mechanism has been suggested for the oxidative degradation of duckweed [54,56]. More detailed discussion of the mechanism of oxidative pyrolysis of biomass can be found in [84].

#### Distribution of biomass pyrolysis products

**Figure 9:**
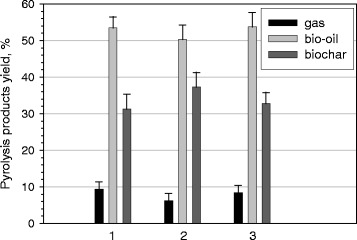
**Distribution of pyrolysis products.** 1) *C. protothecoides*, 2) *A. fumigatus*/*C. protothecoides*; 3) *A. fumigatus.*

Figure 9 shows the distribution of pyrolysis products (biogas, bio-oil, bio-char) for all three biomass samples tested at the pyrolysis temperature of 500°C. The results obtained indicate that within the experimental margin of error, the bio-oil yields for all samples were similar. Pyrolysis of the samples generate on average 6% to 9% gas, 30% to 38% solid and 50% to 55% bio-oil fractions. No significant differences can be observed in the proportion of pyrolytic products between *C. protothecoides*, *A. fumigatus* and *A. fumigatus/C. protothecoides*. The production of the main pyrolytic products from *C. protothecoides* correlates well with production of bio-oil, biogas and bio-char from microalgal samples reported earlier [52]

The composition of the pyrolysis gas is shown in Table 4. The major gaseous products of algal biomass pyrolysis were CO_2_, CO and CH_4_ along with small amounts of H_2_ and C_2_+ hydrocarbons. CO_2_ is by far the major component of the pyrolysis gas produced from all samples, amounting to about 70 to 85 vol.% of the gas. It can be seen from the Table 4 that pyrolysis of *C. protothecoides* produces a higher percentage of H_2_ and a correspondingly lower percentage of CO, compared with other samples. This could be attributed to the water-gas shift reaction taking place in the presence of the catalytically active mineral components of the algae. *C. protothecoides* shows the highest content of gaseous hydrocarbons (C_1_ to C_4_) and the lowest CO_2_ content among all the samples tested.

**Table 4:** **Composition of gaseous products pyrolysis in vol. %**

**Species**	**H**_**2**_	**CO**_**2**_	**CO**	**Methane**	**Ethane + ethene**	**Propane + propene**	**Total C**_**4**_
*C. protothecoides*	2.1	69.6	5.8	9.7	5.3	4.2	3.3
*A. fumigatus/C. protothecoides*	0.2	84.6	7.4	2.8	1.6	1.6	1.7
*A. fumigatus*	0.6	72.2	17.3	5	2.1	1.3	1.5

Total C4: butane + *iso*-butane + butenes.

#### Analysis of the bio-oil products of pyrolysis

**Figure 10:**
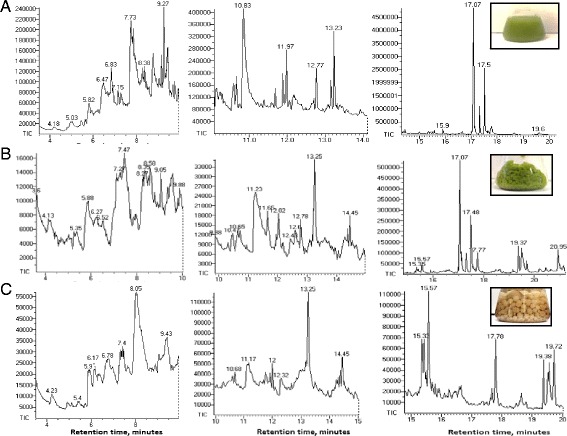
**GC spectra of dichloromethane-dissolved bio-oil samples produced from: (A)** ***C. protothecoides,*** **(B)** ***A. fumigatus*** **/** ***C. protothecoides*** **pellets;** **(C)** ***A. fumigatus.***

The bio-oil produced by pyrolysis of the tested microalgal biomass represented a dark brown-to-black viscous liquid with a pungent odor. Similar observations have been reported by [82]. GC chromatograms of dichloromethane-dissolved bio-oil samples produced from *C. protothecoides*, *A. fumigatus* and *A. fumigatus/C. protothecoides* and retention times of the peaks of the individual components of bio-oils are shown in Figure 10. The peak assignments and the list of molecules identified by the search-match feature of the MS software are summarized in Table 5. Only those peaks with a high degree of certainty, over 90%, are included in this list. The bio-oils produced from *C. protothecoides*, *A. fumigatus* and *A. fumigatus/C. protothecoides* contained a mixture of low-to-intermediate molecular weight compounds representing the derivatives of aromatic and non-aromatic heterocyclic, oxygenated and N-containing compounds such as phenols, pyrroles, indolizines, furanes, indoles, piperidines and others. Many of these compounds were previously identified in pyrolysis products of other micro- and macro-algae representatives, for example, *L. minor,* azolla and others [39,53-56]. The analyzed bio-oil samples showed the presence of long-chain saturated alkanes (paraffins) such as dodecane, tridecane, tetradecane, pentadecane, hexadecane, heptadecane and octadecane along with mono-unsaturated alkanes and other alkane derivatives sizing from C14 to C20, for example, 8-heptadecene, phytol and others. It should be noted that similar long-chain lipid-derived alkanes were found in the pyrolysis products of some algal strains [85,86]. Compounds like 5-methyl-2-furan methanol, 3,4,5-trimethyl-2-cyclopentene-1-one and other oxygenates could be of a carbohydrate origin. Long chain acids and esters (oleic acid, linolenic acid methyl ester, 11,13-dimethyl-1,2-tetradecen-1-ol-acetate) can be attributed to the lipid content of the biomass samples.

**Table 5:** **GC analysis of bio-oil products of** ***C. protothecoides, A. fumigatus/C. protothecoides*** **and** ***A. fumigatus***

**RT, min**	***C. protothecoides***	***A. fumigatus/C. protothecoides***	***A. fumigatus***
4.9		3-Methyl furan and/or 3-cyclopenten-1,2 diol	3-Methyl furan and/or 3-cyclopenten-1,2 diol
5.03		1-(2-Furanyl) ethanone	1-(2-Furanyl) ethanone
5.2			Tetrahydro-4H-pyran-4-ol
5.35		3-Methyl 1H-pyrrole	
5.72		2,4,5-Trimethyl 1H-imidazole	
5.8		4-Methyl piperidine	
5.82			Aconitic anhydride
6.27		2,3-Dimethyl 1H-pyrrole	
6.33	Phenol	Phenol	Phenol
6.52		2-Carboxaldehyde-1H-pyrrole	
6.72	2,3-Dimethyl-1H-pyrrole		
6.83			Cis-4-methyl cyclohexane methanol
7.07	3-Methyl-N (3-methyl butylidene)-1-butanamine		
7.13	4-Ethyl-2-methyl pyrrole		
7.15		5-Methyl-2-furan methanol	5-Methyl-2-furan methanol
7.27		4-Methyl piperdine	4-Methyl piperdine
7.45	4-Ethyl-2-methyl pyrrole	4-Ethyl-2-methyl pyrrole	
7.38	2-Methyl phenol	2-Methyl phenol	2-Methyl phenol
7.9	3-Methyl phenol	3-Methyl phenol	3-Methyl phenol
7.92	4-Ethyl-2-methyl pyrrole		
8.3	3-Ethyl-2,4-dimethyl-1H-pyrrole	3-Ethyl-2,4-dimethyl-1H-pyrrole	
8.58		3-Ethyl-2,5-dimethyl-1H-pyrrole	
8.38	2-Acetyl cyclopentanone		
8.47	4-Ethyl-2, 3-dimethyl -1H-pyrrole		
8.77	4-Methyl phenol	4-Methyl phenol	
	2-Ethyl-3, 4, 5-trimethyl-1H-Pyrrole		
9.28		Dodecane	Dodecane
9.33		4-Amino phenol	
9.43		3-Ethyl-2,5-dimethyl-1H-pyrrole	3-Eethyl-2,5-dimethyl-1H-pyrrole
9.45		1,2,2,3-Tetramethyl cyclopent-3-enol	1,2,2,3-Tetramethyl cyclopent-3-enol
9.67	4-Ethyl phenol	4-Ethyl phenol	
9.87	1, (3-Aminopropyl)-2-pyrrolidone	1,(3-Aminopropyl)-2-pyrrolidone	
9.73	Nanofin (2,6-dimethyl piperidine)		
10.13	2-Ethyl phenol		
10.67		2-Methyl-2-ethyl-pyrrolidine	
10.67	3, 4, 4-Trimethyl-cyclopenten-1-one	3,4,5-Trimethyl-2-cyclopentene-1-one	
10.67		Tridecane	Tridecane
10.71			Indole/benzene nitrile
10.83	Indole	Indole	
11.57	1, 2-Dihydro-1, 1, 6-trimethyl naphthalene	1,2-Dihydro-1,1,6-trimethyl naphthalene	
11.77	Indolizine	Indolizine	
11.98	Tetradecane	Tetradecane	Tetradecane
12.13	2-Methyl indole	2-Methyl indole	
12.78			7-Methyl indolizine
12.79	2, 6, 10-Trimethyl dodecane	2,6,10-Trimethyl dodecane	
12.83	3-Methyl indolizine		
13.23	Pentadecane	Pentadecane	Pentadecane
13.37	4-Methyl 1H-indole		
14.43	Hexadecane	Hexadecane	Hexadecane
15.38		8-Heptadecene	8-Heptadecene
15.57		Heptadecane	Heptadecane
15.93	3,7,11,15-Tetramethyl-2-hexadecene	3, 7, 11, 15-Tetramethyl-2-hexadecene	
16.65		Octadecane	Octadecane
16.75	9-Nonadecene	9-Nonadecene	
17.1 to 17.55	Isomers of 3,7,11,15-tetramethyl-2-hexadecen-1-ol (phytol)	Isomers of 3, 7, 11, 15-tetramethyl-2-hexadecen-1-ol (phytol)	
17.8		Oleic acid	Oleic acid
18.25		Palmitic acid	Palmitoleic acid
18.65	Cis-9-eicosen	Cis-9-eicosen	
19.38			Pentadecane-2, 4-dione
19.58	Heneicosane	Heneicosane	
19.85		11,13-Dimethyl-1,2-tetradecen-1-ol-acetate	
19.75		Linolenic acid methyl ester	Linolenic acid methyl ester

Phytol (3,7,11,15-tetramethyl-2-hexadecen-1-ol), the product of the degradation of chlorophyll, represents the most abundant pyrolysis product in *C. protothecoides* and *A. fumigatus/C. protothecoides.* Phytol is a commercially important product used in the manufacturing of synthetic forms of vitamin E [87] and vitamin K1 [88], as well as in the production of cosmetics, fragrances, shampoos, soaps, detergents and household cleaners [89]. Because of the absence of chlorophyll molecules, phytol is not present in the bio-oil produced from *A. fumigatus.* Another difference between *C. protothecoides* and *A. fumigatus* bio-oils is that the latter has a relatively higher content of straight chain hydrocarbons and fewer nitrogen-containing compounds.

Of particular practical importance is the conversion of bio-oil to petroleum-equivalent transportation fuels. Long-chain saturated alkanes of diesel range, such as C13 to C17 paraffins found in bio-oils, can be directly added to commercial diesel fuel; for reviews, see [7-9,13-15,76,90]. Since the physical properties of phytol and its fuel characteristics (for example, density, cetane number and heat of combustion) are close to that of diesel fuel, it is currently being investigated by the US Argonne National Laboratory researchers as a potential drop-in biofuel (http://www.transportation.anl.gov/engines/multi_dim_model_biofuels.html). Most of the oxygenated products identified in the pyrolysis oils can potentially be converted into renewable (or ‘green’) gasoline and diesel fuels using existing and emerging techniques, such as catalytic hydrodeoxygenation (CHDO) and hydrotreatment. In the CHDO process, the oxygenated compounds of bio-oils at moderate temperatures (350°C to 400°C) in the presence of catalysts (typically alumina, silica or carbon-supported Ru, Pd, Ni and Co) are transformed into compounds with low-oxygen content that can be injected in the specific points of a refinery chain. N- and S-containing compounds (that are poisons for many industrial catalysts) present in the bio-oils could be dealt with conventional hydrodesulfurization and hydrodenitrification processes that are used at modern refineries.

In a recent paper [49], the authors described hydroprocessing of microalgae-derived bio-oil over HZSM-5 catalyst at 400°C to 500°C under 4.35 MPa pressure of hydrogen. It was found that hydrotreatment greatly reduced the heteroatom (O, S, N) content of the bio-oil. For example, the sulfur content was reduced to less than 0.1 wt.%, and the O/C ratio was reduced by one order of magnitude. Catalytic hydroprocessing of the bio-oil resulted in paraffinic oil, 95% of which consisted of carbon and hydrogen, which made it useful as a feedstock for production of liquid transportation fuels. This study demonstrated the opportunities to engineer the composition of the products obtained from algal bio-oil.

#### Analysis of bio-solids

Algal biomass is a potential resource for the production of value-added bio-solids such as bio-chars and inorganic ashes (the latter is produced by combustion of either bio-char or raw algae biomass). Plant-derived bio-char has been known for centuries as a soil-amending agent that can greatly enhance crop growth by improving moisture retention and nutrient holding capacity of soils. Bio-char has also been used as a precursor for the production of activated carbons (AC) and other carbonaceous products [91]. Due to availability, chemical inertness (for example, resistance to sulfur, phosphorus and nitrogenous compounds) and low cost AC are widely used as adsorbents and catalyst supports; and in some cases, they are utilized directly as catalysts. For example, we have previously reported that bio-char exhibited appreciable catalytic activity in biogas reforming with the production of syngas [56].

Additional file 7 shows the photographs and SEM images of bio-char and ash obtained from the tested samples. Based on the TGA/DTG analyses of biomass samples (see Table 3), *C. protothecoides* showed a higher content of fixed carbon and ash compared to other samples. In contrast, *A. fumigatus* showed lowest yields of bio-char and ash. The inorganic ash content of *C. protothecoides* (4.1 wt.%) exceeds that of plant biomass (typically, 1 to 2 wt.%) [51,52]. *C. protothecoides* ash presents a white amorphous powder (Additional file 7C). SEM image of *C. protothecoides* ash (Additional file 7D) shows the presence of one to three micron-size particles with some larger particles scattered over the surface.

Additional file 8 shows the data on energy-dispersive spectroscopic (EDS) analysis of the ash and bio-char products of pyrolysis of all biomass species tested. It can be seen that phosphorus is the major component of *C. protothecoides* ash. Phosphorus is also present in the bio-chars produced from *A. fumigatus/C. protothecoides* and *A. fumigatus. C. protothecoides* ash also contains K, Ca, Mg and small amounts of Al, Fe and other elements, which points to the significant salt uptake capacity of *C. protothecoides*. Bio-chars produced from *A. fumigatus/C. protothecoides* and *A. fumigatus* samples (besides the main component - carbon) contain K, N, P and an appreciable amount of sulfur.

It should be noted that the presence of inorganic compounds in the algal biomass in relatively large quantities may provide a certain catalytic effect on the thermal degradation processes (both in inert and oxidizing atmosphere), thus reducing the pyrolysis temperature. It has been reported, for example, that decomposition of cellulose is catalyzed by inorganic compounds present in biomass [92].

#### Microalgal/fungal biomass conversion to bio-oil via extraction and pyrolysis technologies

In our study, we analyzed two technological approaches to recover bio-oil from algae, fungi and fungal-algal pellets: (i) conventional extraction with organic solvents which gave 10% to 26% of TAG-containing neutral lipids which can be converted into biofuel using TE technology and (ii) pyrolysis, which produced about 50% of DW as bio-oil which can be further converted into petrochemicals and liquid fuels using CHDO and/or hydrocracking techniques. The extraction processes, in general, are very energy intensive and costly since they involve expensive solvents and significant consumption of electricity. In addition, many extraction techniques require highly concentrated substrates (which sharply increases energy consumption). Among other challenges facing the extraction processes is the presence of large amounts of moisture (which can participate in side hydrolysis reactions) and undesirable components such as chlorophyll and non-transesterifiable lipids. On the positive side, once extracted, the oil can be quantitatively transformed into biodiesel via commercially mature TE technology with the yields in excess of 98% (although, the use of algal oil in this process is a relatively new area) [93].

Thermochemical pathways for conversion of microalgae and fungi to biofuels present a viable alternative to extraction technology. The options primarily include: (i) pyrolysis, (ii) gasification coupled with Fischer-Tropsch synthesis, and (iii) hydrothermal liquefaction. The detailed overview of these technological approaches to algae-based biofuel production was recently published by Muradov (2013, 2014) [94,95]. The pyrolysis of microalgae and fungi into biofuels could benefit from reduced costs associated with the elimination of a number of steps, such as product extraction and pre- and post-processing. An additional advantage of the thermochemical approach stems from the fact that it is amenable to processing of a broad and diverse range of microalgal and fungal strains, even ones with tough cell walls, which could substantially expand the feedstock supply.

The pyrolysis route, however, is facing several challenges of technical and economic nature. The hydrotreatment processes require substantial amounts of hydrogen and the use of expensive catalysts, which may add to the cost of the final product. In most cases, algal and fungal feedstocks have very high moisture content, which might require an energy-intensive upstream dehydration step for the process to operate efficiently. The presence of phosphorus, nitrogenous and sulfurous compounds in the bio-oil could be detrimental to conventional hydrogenation or hydrocracking catalysts. Finally, the significant fraction of bio-oil could be lost during hydrotreatment processes due to undesirable side reactions. Thus, in order to efficiently and cost-effectively process algae and fungi to liquid biofuels via pyrolysis, it would be necessary to optimize the process on several levels, including the purification and stabilization of oil, the improvement of the catalysts tolerance toward the contaminants and the enhancement in the selectivity of the hydrotreatment processes.

Based solely on algae/fungi-to-biofuel yield criterion, the feasibility of biomass conversion to liquid biofuels via extraction-TE *versus* pyrolysis-CHDO routes will be determined (among other factors) by its lipid content. In our study, we have not optimized conditions for high lipid production in *C. protothecoides.* Other studies demonstrated that the crude lipid content of *C. protothecoides* grown under heterotrophic conditions could reach up to 55% of lipid/DW [52]. The same work also showed that *C. protothecoides* cells grown under heterotrophic conditions produced 3.4 times more pyrolysis bio-oil than when grown photoautotrophically. Oleaginous fungi, such as *M. circinelloides* with lipid concentration up to 25% of DW, can be used for microalgal flocculation contributing to total lipid production. Detailed techno-economic evaluation of both approaches would be necessary to make a final determination of their economic feasibility and commercial viability.

The described technology of microalgal flocculation by fungal cells is still primarily based only at the lab scale. Further, extensive techno-economic analysis is now required to make this technology economically feasible at a larger scale. Key areas of this research include the following: (i) the application of biologically safe fungal strains and ecotoxicological studies together with risk assessment of the technology; (ii) assessment of self-pelletization and co-pelletization efficiencies; (iii) reduction of the cost of growth nutrients through the application of alternative sources of carbon, nitrogen and phosphorus; (iv) establishment of efficient and cheap downstream processing technologies which include oil extraction from fungal/fungal-algal cells and their conversion to fuels.

## Conclusions

The described fungal-assisted microalgal flocculation shows a potential to solve a number of key challenges that algal biotechnology is facing, in particular:

(i) *Efficient harvest of freshwater and seawater microalgae. A. fumigatus* showed efficient harvest of *C. protothecoides* and *T. suecica* strains widely used by research groups and commercial companies for biodiesel and value chemical production.
(ii) *Enhancement of oil production and optimization of its composition.* Fungal-algal co-pelletization showed a synergistic effect on total biomass and lipid production. The composition of fatty acids can be tailored and optimized through co-cultivating different algae and fungi without the need for genetic modification.
(iii) * Energy-efficient and potentially cost-effective oil extraction from microalgal cells.* Cell disruption is an important step in biofuel production since microalgal cell walls are generally thick and consist of multiple layers. Generation of cell wall-free algal protoplasts after co-cultivation with fungal cells can significantly reduce the cost of oil extraction.
(iv) *Alternative carbon, nitrogen, phosphorus and microelements from waste biomass as a sustainable and renewable nutrient supply. A. fumigatus* pellets grown on wheat straw showed high algal flocculation rates. Fungal/algal co-pellets showed a synergistic effect on the bioremediation of swine wastewater. Growing fungal and algal cells on alternative sources of carbon and N/P and subsequent wastewater filtration can potentially improve the economics of large-scale algal biotechnology. Dilution of wastewater with seawater for treatment with *A. fumigatus/T. suecica* pellets reduced the amount of freshwater required, thereby making the whole process more economic.
(v) *Sustainable bioenergy/biofuel production*. Because of their high growth rate, high lipid yield and the composition of essential fatty acids, both components of the system, microalgae and fungi, can be used as feedstocks for the production of sustainable and renewable bioenergy/biofuels. In combination with the widely used TE-based biodiesel production, generated biomass can be thermochemically (via pyrolysis) converted into biogas, bio-solids and a range of glycerol-free petrochemicals including straight-chain C12 to C21 alkanes that can be directly used as the blending components of biodiesel. Since fungal chitin containing cell walls represent a formidable challenge for conventional chemical and biochemical oil-extraction methods, pyrolysis represents an attractive method for production of value-added chemicals from the fungal cells.

## Methods

### Fungal isolation

Fungal representatives were isolated from compost, straws and soil as a rich source of fungi. These samples were kept in ziplock plastic bags and stored at −20°C for further investigation. Selective media (BH medium) [96] was used as the basis for fungal isolation. The collected samples were serially diluted (10:1 to 10:6) using phosphate buffer saline (0.1 M) under, and an aliquot (150 μl) of each dilution was spread onto BH agar plates. These plates were incubated for 6 days at 30°C and 55°C for mesophilic and thermophilic fungi, respectively. For fungal isolation, an antibiotic solution of 0.015 g/L of tetracycline (dissolved in sterilized Milli-Q water, filtered through a sterile 0.22-μm filter) was added to the media. Following isolation, fungi were re-streaked until purified. All cultures were stored at −80°C with 25% of glycerol.

### Preparation for seed fungal spores

For activation, the stored spores were grown at 25°C for 5 days on plates with potato dextrose broth (PDB) containing 20 g/L glucose. Sterile water (10 mL) was added to harvest the spores, and the spore solution was used as the inoculation for the co-culture after the number of spores in the solution was counted.

### Preparation of fungal pellets

To achieve pelletization spore solutions (1.5 to 2.0 × 10^7^ spores/L) were cultivated at 28°C in PDB or in carbon-free media (3 g/L peptone, 0.6 g/L KH_2_PO_4_, 0.001 g/L ZnSO_4_, 0.4 g/L K_2_HPO_4_, 0.005 g/L FeSO_4_, 0.5 g/L MnSO_4_, 0.5 g/L MgSO_4_) supplemented with 1% TWS, with a shaking speed of 150 rpm for 72 h. The spore solutions were then cultured at 28°C with a shaking speed of 150 rpm for 72 h to achieve pelletization. Before mixing with microalgal cultures, pellets were washed twice in sterile water, followed by sterile algal media.

### Acid pretreatment of wheat straw

One gram of fine powder (approximately 1 mm sin size) of dry wheat straw was mixed with 1 M sulphuric acid and autoclaved for 10 min at 121°C, allowed to cool, filtered through Whatman No.1 filter paper, then washed 10 times with sterile water followed by 0.1 M sodium hydroxide. The powder was dried at 80°C and added to the media to a final concentration of 1%.

### Screening of fungal strains for oil content

Screening of the fungi for oil yield was carried out using a slightly modified method described by [62]. Fungi were grown in Erlenmeyer flasks containing 50 mL of enrichment broth, EB [100 g/L glucose, 1 g/L yeast extract, 2 g/L potassium dihydrogen phosphate (KH_2_PO_4_), 0.75 g/L magnesium sulfate (MgSO_4_), 1 g/L di sodium hydrogen phosphate (Na_2_HPO_4_), 0.2 g/L calcium chloride, 0.01 g/L iron chloride, 0.1 g/L zinc chloride] in an orbital mixer/shaker at 25°C for 3 days. After 3 days, a 5 mL sample was taken from each flask and spun in a centrifuge at 5,000 rpm for 10 min and washed twice with sterile water. One fungal pellet was placed in the centre of a carbon-deficient agar (same as EB, no glucose, plus 15 g/L agarose). The growth area was marked and scanned at times, 0, 12 and 24 h. For relative growth rate analysis, the diameter of the final fungal growth after 12 and 24 h were divided by the diameter of the initial fungal inoculum (time 0).

### Microalgal strains

Strains were obtained from a Culture Collection of Algae at the University of Texas at Austin (UTEX, http://web.biosci.utexas.edu/utex/). *C. protothecoides* was grown axenically under mixotrophic conditions (3% glucose, light) in the media described by UTEX. *T. suecica* was grown autotrophically in F2 media suggested by UTEX. Under mixotrophic and autotrophic conditions, cultures were grown in related media under constant light (200 μmol m^−2^ s^−1^), shaking at 150 rpm and 25°C. Growth rates were analyzed by (i) counting the cell numbers using a TC10™ Automated Cell Counter (BioRad, Hercules, CA, USA), (ii) by measuring OD_540_ for *C. protothecoides* and OD_750_ for *T. suecica*, (iii) by concentration of chlorophyll A + B using a POLARstar Omega Multi-Mode Microplate Reader with Fluorescent Polarization (BMG LABTECH, Ortenberg, Germany) and by biomass production. Chlorophyll was extracted with ethanol, and absorbance at 649, 665, and 750 nm was determined. Chlorophyll concentration (μg/mL) was calculated using the equation Chl (μg/mL) = [6.1x (E665 to E750) + 20.04 (E649 to E750)], K, where E is extinction at the corresponding wavelength; K is the dilution factor and 6.1 and 20.04 are extinction coefficients [97]. For biomass analysis, microalgal cultures were centrifuged at 6,000 g and then was washed twice with sterilized water and centrifuged again and dried at 65°C.

### Nile red staining

For Nile red staining, the algal cells, fungal cells and co-cultivated pellets were collected by centrifugation and re-suspended in 1 mL of 20% DMSO containing 5 μL of Nile red stock solution (0.10 mg/mL of Nile red dissolved in acetone) and incubated at 50°C with shaking at 150 rpm for 5 min. The stained pellets were then subjected to fluorescence microscopy to observe the formation of lipid droplets in co-cultivated cells using a Leica DM 2500 with an attached camera (Leica DFC 310 FX). Nile red filter has excitation at 543 nm and emission at 555 to 650 nm.

### Co-cultivation

For the 12-well microtitre plate experiments, fungal pellets grown on PDB were added to 4.5 mL of suspensions of *C. protothecoides* (grown mixotrophically, 1.5 × 10^7^ cells/mL) and *T. suecica* (grown autotrophically, 6.5 × 10^6^ cells/mL), and mixtures were shaken at 100 rpm for 24 h. Microalgal cell number, OD_540_ and OD_750_ and chlorophyll concentrations were analyzed at time 0 and 24 h. For 250 mL experiments, the fungal pellets were produced in PDB or 1% TWS for 3 days, excess liquid was removed using a Pasteur pipette and the pellets were washed with sterile water followed by algal medium. Algal culture (250 mL) was added (*C. protothecoides*, 1 to 3 × 10^9^ cells/mL grown mixotrophically; *T. suecica*, 7 to 12 × 10^8^ cells/mL, grown autotrophically). The mixtures were shaken at 150 rpm for 48 h under constant light (200 μmol m^−2^ s^−1^) at 25°C. Fungal and algal mono-cultures were also grown for 48 h as controls. All of the experiments were biologically replicated at least three times. Cell number, biomass, OD_540/750_ and chlorophyll concentrations were measured at time 0 and 48 h. Algal samples were analyzed 3 min after stopping rotation. Flocculation efficiency (FE) was calculated based on changes in OD, cell numbers and chlorophyll concentrations of uncaptured algal cells in the co-cultivation media at time 0 and 24/48 h later according to the following formula: $\mathrm{F}\mathrm{E}\%=\frac{A-B}{A}\times 100$, where *A* = OD, cell number and chlorophyll concentration at time 0; *B* = OD, cell number and chlorophyll concentration after 24 or 48 h. The morphology of the fungal and algal cells and co-cultivation pellets was observed under bright field conditions using a Leica DM 2500 with an attached camera (Leica DFC 310 FX, Solms, Germany).

### Genotyping of fungal strains

The identification of the fungal strain was based on nucleotide sequence analysis of the internal transcribed space (ITS) region. Genomic DNA was extracted as described by [62,69]. The ITS1 region was amplified by PCR with primers ITS1: TCCGTAGGTGAACCTGCGG and ITS2: GCTGCG TTCTTCATCGATGC [69]. Satisfactory 16S rDNA sequences were codon aligned and compared with published reference strains in the National Centre of Biotechnology Information. An agreement between the query and reference sequences of more than 95% denoted a positive match. Confirmatory phylogenetic reconstruction was also performed using standard bioinformatics software such as the PAUP (Sinauer Associates Inc., Sunderland, MA, USA).

### Wastewater treatment

The swine lagoon wastewater was provided by Dr J Hill, Termes Consulting Ltd, Melbourne. Swine wastewater was treated anaerobically. Wastewater samples were centrifuged to remove large particles, filtered through Whatman filter paper and autoclaved at 121°C, allowed to cool to room temperature and stored at 4°C. The concentrations of NH_4_^+^-N and PO_4_^−3^-P in the ASW were 680.7 and 145.7 mg/L, respectively. The concentration of other inorganic nitrogen in the wastewater, such as NO_3_^−^-N, was very low and not reported. Wastewater was diluted to 25% with tap water for experiments with *C. protothecoides* and sea water for experiments with *T. suecica.* The fungal/algal pellets produced on PDB were harvested by filtration, and 200 wet pellets were added to the 250 mL of wastewater (approximately, 1 g/L DW). The mixtures were shaken at 150 rpm for 48 h. Samples of growth media were analyzed for ammonia cations, nitrate and phosphate anions using an ion chromatography system Dionex ICS-1100 (Thermo Scientific, Waltham, MA, USA).

### Lipid yield and fatty acid profile analysis

Extraction and analysis of lipid yield and FAME composition analysis of algal, fungal and fungal-algal pellets were performed using a method previously described [98,99].

### Biomass pyrolysis experiments

The experimental setup for biomass pyrolysis consists of a quartz tube reactor with the outside and inside diameter of 12 and 10 mm, respectively. A sample of algal biomass (pre-dried at 110°C overnight) (2.0 ± 0.1 g) was placed inside the reactor. Prior to each run, the reactor and all connecting lines were purged with ultra-pure Ar (99.999%). The flow rate of Ar sweep gas was set at a constant flow rate of 100 mL/min using a metering valve and a calibrated rotameter. Heating of the quartz tube reactor was carried out using a temperature-controlled tube furnace (Omega Engineering, Stamford, CT, USA). Two thermocouples (one external to the tube and one internal) were used to monitor the reactor temperature during pyrolysis. The furnace and the quartz tube were vertically aligned, so that the liquid products dripped into the condenser assembly, which was chilled using ice. After condensation of the liquid product, the gas passed through a glass wool filter before being collected in a gas sampling Teflon bag. The condenser was weighed before and after the reaction to determine the amount of the liquid product collected. The solid product (bio-char) of pyrolysis was collected and weighed.

### Analysis of biomass pyrolysis products

#### Gaseous product analysis

The pyrolysis gas analysis was performed using a Varian 450 gas chromatograph (GC) with thermal conductivity detector (TCD) for permanent gases (H_2_, air, CH_4_, CO, CO_2_) and flame ionization detector (FID) for hydrocarbon gases. Argon was used as a carrier gas, and three columns were used for GC separation: PLOT alumina/KCl, molecular sieve 5A and Haysep Q.

### Liquid product analysis

The collected biomass pyrolysis liquid product (bio-oil) was dissolved in aliquot amounts of dichloromethane (DCM) and injected into the Agilent 6890 N (Agilent Technologies, Santa Clara, CA, USA) coupled to a JEOL GCMate-II (JEOL, Akishima-shi, Tokyo, Japan) gas chromatograph-mass spectrometer (GC-MS). Typical (GC-MS) parameters used in the bio-oil analyses were as follows: carrier gas He flow of 2 mL/min, column: HP-5ms (60 m × 0.32 mm × 0.25 mm), injection port temperature: 300°C, GC interface temperature: 250°C, ion source temperature: 250°C, sample injection volume: 5 μL, split ratio: 50:1. The peaks in the chromatograms were identified using the search-match function in the MS software.

### Thermogravimetric analysis

The thermogravimetric (TGA) and derivative thermogravimetric (DTG) analyses of the biomass samples were carried out using a Perkin-Elmer Diamond TG/DTA instrument with helium or ultra-zero air as carrier (sweeping) gases at the flowrate of 200 mL/min. In a typical TGA/DTG analysis, about 12 mg of the sample was loaded into the instrument and heated from 25°C to 950°C at the heating rate of 20°C/min in helium (pyrolysis mode) or air (combustion mode). The dry biomass samples were used as is (without additional drying). TGA, run using He as carrier gas was used to determine the moisture, volatiles and fixed carbon contents, whereas TGA with air as a carrier gas was used to determine the ash content of biomass samples. The ash content of the sample was found from the amount of solids remaining at the end of the TGA combustion mode run. The amount of fixed carbon was calculated by subtracting the ash content from the amount of solids remaining at the end of the TGA pyrolysis mode run.

### SEM analysis

SEM analysis was conducted using a Hitachi TM-1000 tabletop scanning electron microscope. Energy-dispersive spectroscopic (EDS) analysis of the bio-char and ash samples was conducted using a Bruker AXS instrument.

### Statistical analysis

All experiments in this study were conducted in triplicate. The experimental data were subjected to one-way analysis of variance (ANOVA) as implemented in the GraphPad InStat 3 statistics platform. Tukey simultaneous tests were conducted to determine the statistical differences between treatments. In order to ascertain that the observed variations in growth rates, efficiency of nutrients uptake and the yield of pyrolysis products were statistically significant, probability (*P*) values were determined. A 95% confidence level (*P* ≤ 0.05) was applied for all analyses.

## Additional files

Additional file 1:
**Lichen phenotypes.** Bar = 10 cm. Additional file 2:
**Table of fungal strains.**
Additional file 3:
**Screening of fungal strains on carbon-free media for oil accumulation.** **(A)** Phenotypic evaluation of fungal strains grown on carbon-free plates; 1) diameter of fungal growth area at time 0; 2) diameter of fungal growth area after 12 h; 3) diameter of fungal growth area after 24 h; **(B)** growth ratio of 33 fungal strains on carbon-free plates. Additional file 4:
**Phenotypic evaluation of fungal pelletization.** **(A)** Fungal representatives grown on PDB; **(B)** *A. fumigatus* grown on PDB. 1) pellets grown at 250 rpm; (2) pellets grown at 150 rpm; 3) pellets grown at 50 rpm. Bar = 1 cm. Additional file 5:
**Microscopic analysis of** ***A. fumigatus*** **-algal** **pellets.** **(A-D)** Cross section of *A. fumigatus/T. suecica* pellet: **(A)** middle part of pellet; (B) between parts A and C; **(C, D)** surface of pellet; **(E**, **F**, **I)** *A. fumigatus/C. protothecoides*; **(G, H, J, K, L, M)** *A. fumigatus/T. suecica.* A-D: 20 μm cryo-sections; **(E-M)**: images of algal-fungal suspensions; A, B, C, E, G: bright-field images; D, F, H, K, L, M: UV light images. red spots represent chloroplast’s fluorescence; (I, J) Nile red staining. Yellow spots represent oil bodies; *CV* cell walls. Scale = 20 μm. Additional file 6:
**Evaluation of microalgal growth in media containing 5% and 20% of** ***A. fumigatus/*** **TWS media.** 1) control; 2) algal growth media containing 5% of *A. fumigatus/*TWS media; 3) algal growth media containing 20% *A. fumigatus/*TWS media. Additional file 7:
**Optical and SEM images of bio-char and inorganic ash.** **(A)** Bio-char from *A. fumigatus/C. protothecoides*; **(B)** bio-char from *A. fumigatus*; **(C)** ash from *C. protothecoides*, **(D)** SEM of ash from *C. protothecoides* (magnification × 2,500). Additional file 8:
**EDS spectra of the ash and bio-chars produced from biomass samples.** **(A)** Ash from *C. protothecoides*; **(B)** bio-char from *A. fumigatus/C. protothecoides*; **(C)** bio-char from *A. fumigatus*. The distribution of elements is presented in atomic percent.

## Abbreviations

AC: activated carbons
*A. fumigatus*: *Aspergillus fumigatus*
Algal: strains
*C. protothecoides*: *Chlorella protothecoides*
*T. suecica*: *Tetraselmis suecica*
ASW: anaerobically digested swine wastewater
EDS: energy-dispersive X-ray spectroscopy
dw: dry weight
DTA: differential thermal analysis
DTG: derivative thermogravimetric analysis
FID: flame ionization detector
GC: gas chromatography
HPLC: high-performance liquid chromatography
LC-MS: liquid chromatography-mass spectroscopy
SEM: scanning electron micrograph
TCD: thermal conductivity detector
TGA: thermogravimetric analysis
TE: transesterification
TWS: acid-treated wheat straw
