# Supplementary figures and images for: Fungal-assisted algal flocculation: application in wastewater treatment and biofuel production
Source: Biotechnol Biofuels. 2015 Feb 15;8:24. doi: 10.1186/s13068-015-0210-6 (PMC4355497; doi:10.1186/s13068-015-0210-6)

## Slide 1
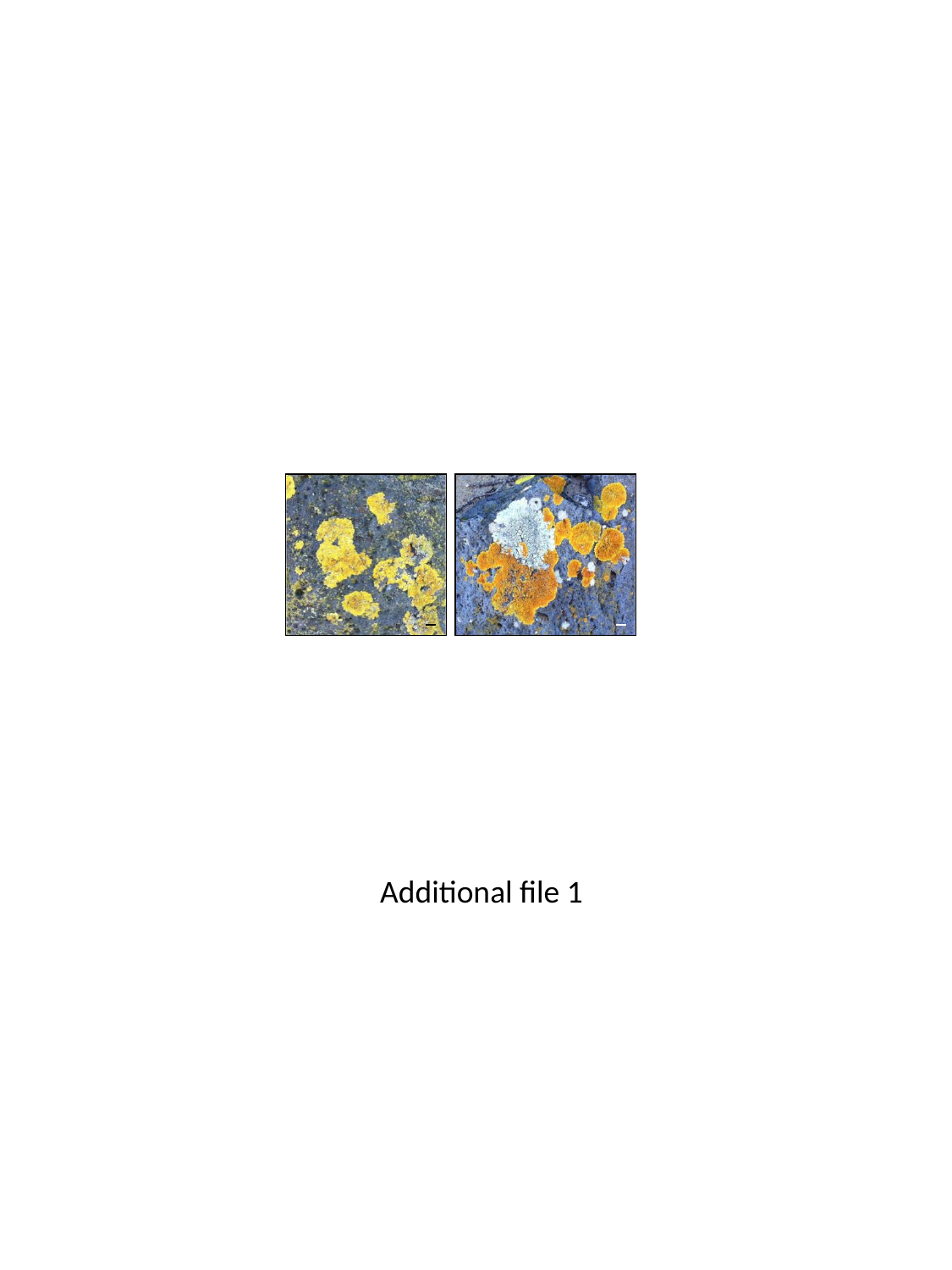

Additional file 1

Supplement: Additional file 1: — Lichen phenotypes. Bar = 10 cm. [file 13068_2015_210_MOESM1_ESM.pptx]

## Slide 1
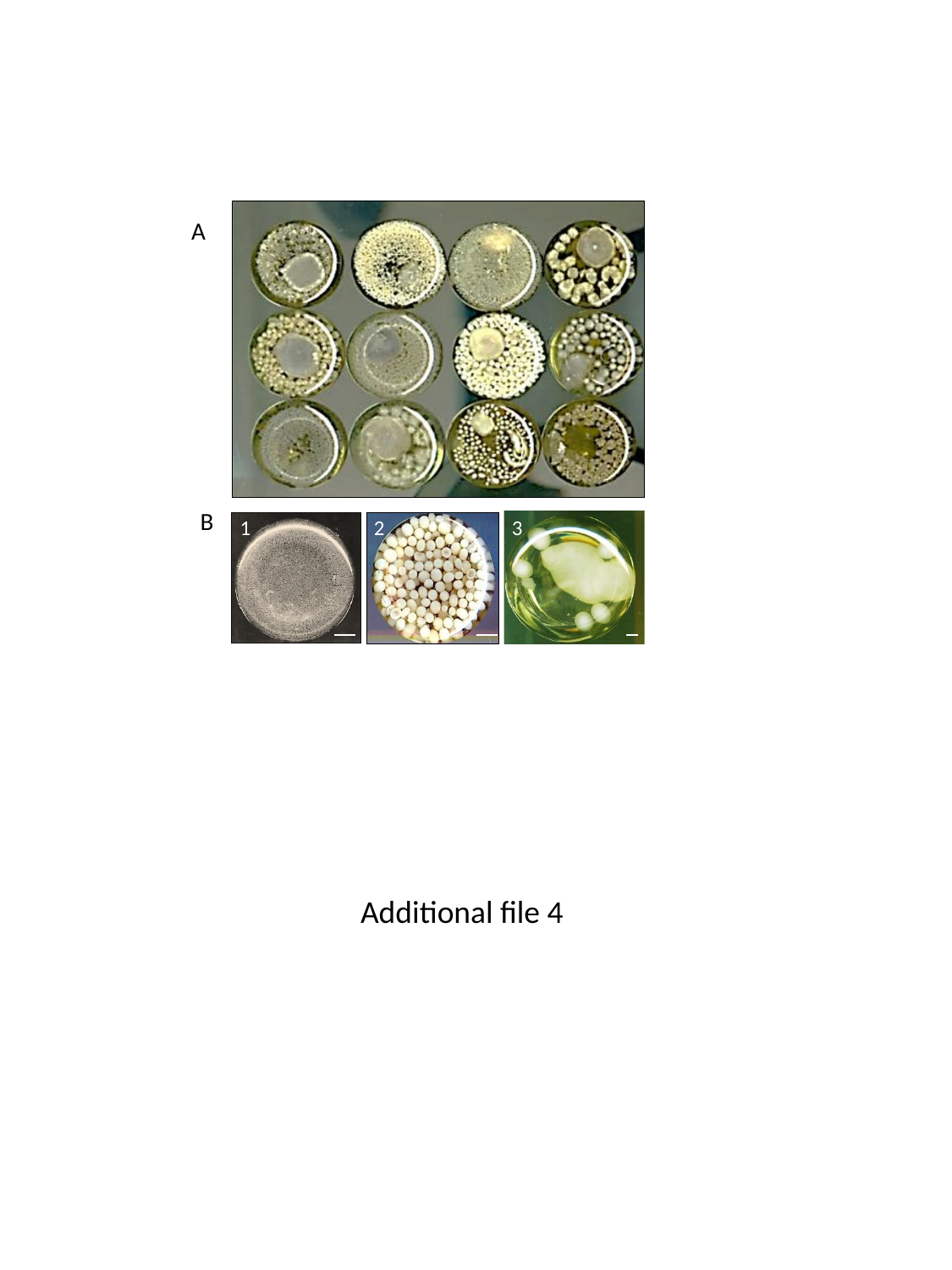

A
a
b
c
d
e
f
B
3
2
1
Additional file 4

Supplement: Additional file 4: — Phenotypic evaluation of fungal pelletization. (A) Fungal representatives grown on PDB; (B) A. fumigatus grown on PDB. 1) pellets grown at 250 rpm; (2) pellets grown at 150 rpm; 3) pellets grown at 50 rpm. Bar = 1 cm. [file 13068_2015_210_MOESM4_ESM.pptx]

## Slide 1
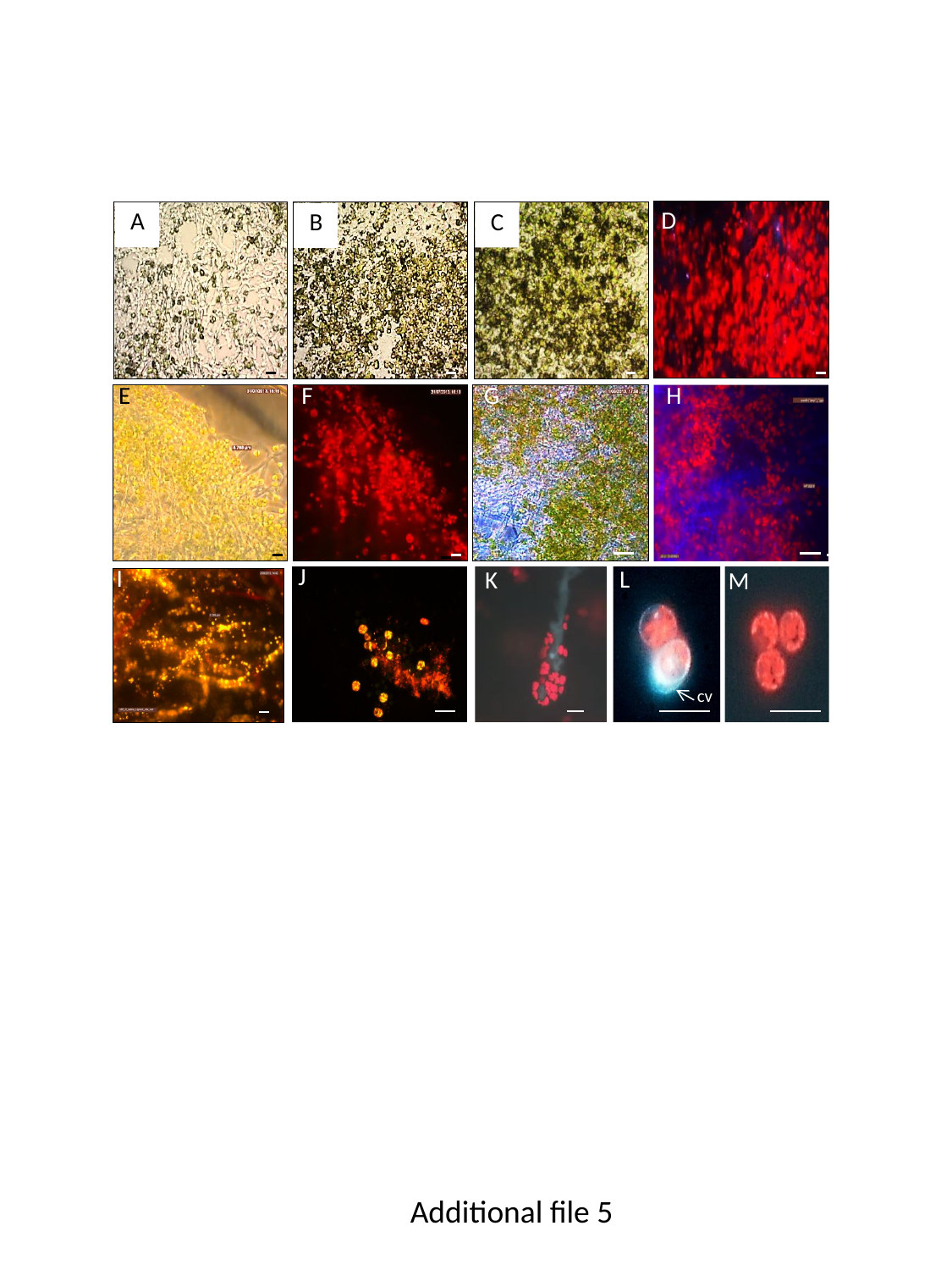

D
A
C
B
E
F
G
H
J
L
I
L
K
M
cv
Additional file 5

Supplement: Additional file 5: — Microscopic analysis of A. fumigatus -algal pellets. (A-D) Cross section of A. fumigatus/T. suecica pellet: (A) middle part of pellet; (B) between parts A and C; (C, D) surface of pellet; (E, F, I) A. fumigatus/C. protothecoides; (G, H, J, K, L, M) A. fumigatus/T. suecica. A-D: 20 μm cryo-sections; (E-M): images of algal-fungal suspensions; A, B, C, E, G: bright-field images; D, F, H, K, L, M: UV light images. red spots represent chloroplast’s fluorescence; (I, J) Nile red staining. Yellow spots represent oil bodies; CV cell walls. Scale = 20 μm. [file 13068_2015_210_MOESM5_ESM.pptx]

## Slide 1
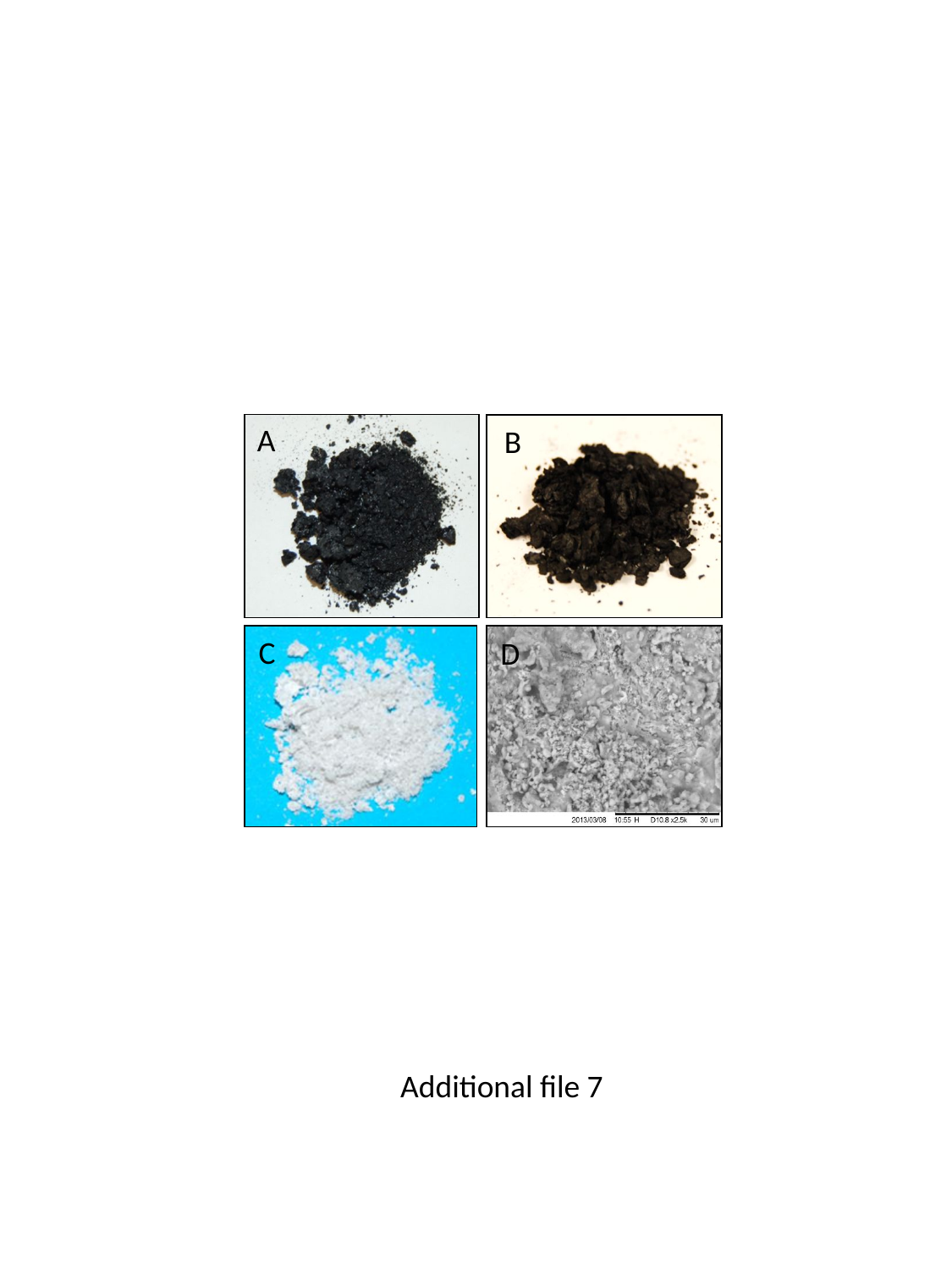

A
B
C
D
Additional file 7

Supplement: Additional file 6: — Evaluation of microalgal growth in media containing 5% and 20% of A. fumigatus/ TWS media. 1) control; 2) algal growth media containing 5% of A. fumigatus/TWS media; 3) algal growth media containing 20% A. fumigatus/TWS media. [file 13068_2015_210_MOESM6_ESM.pptx]

## Slide 1
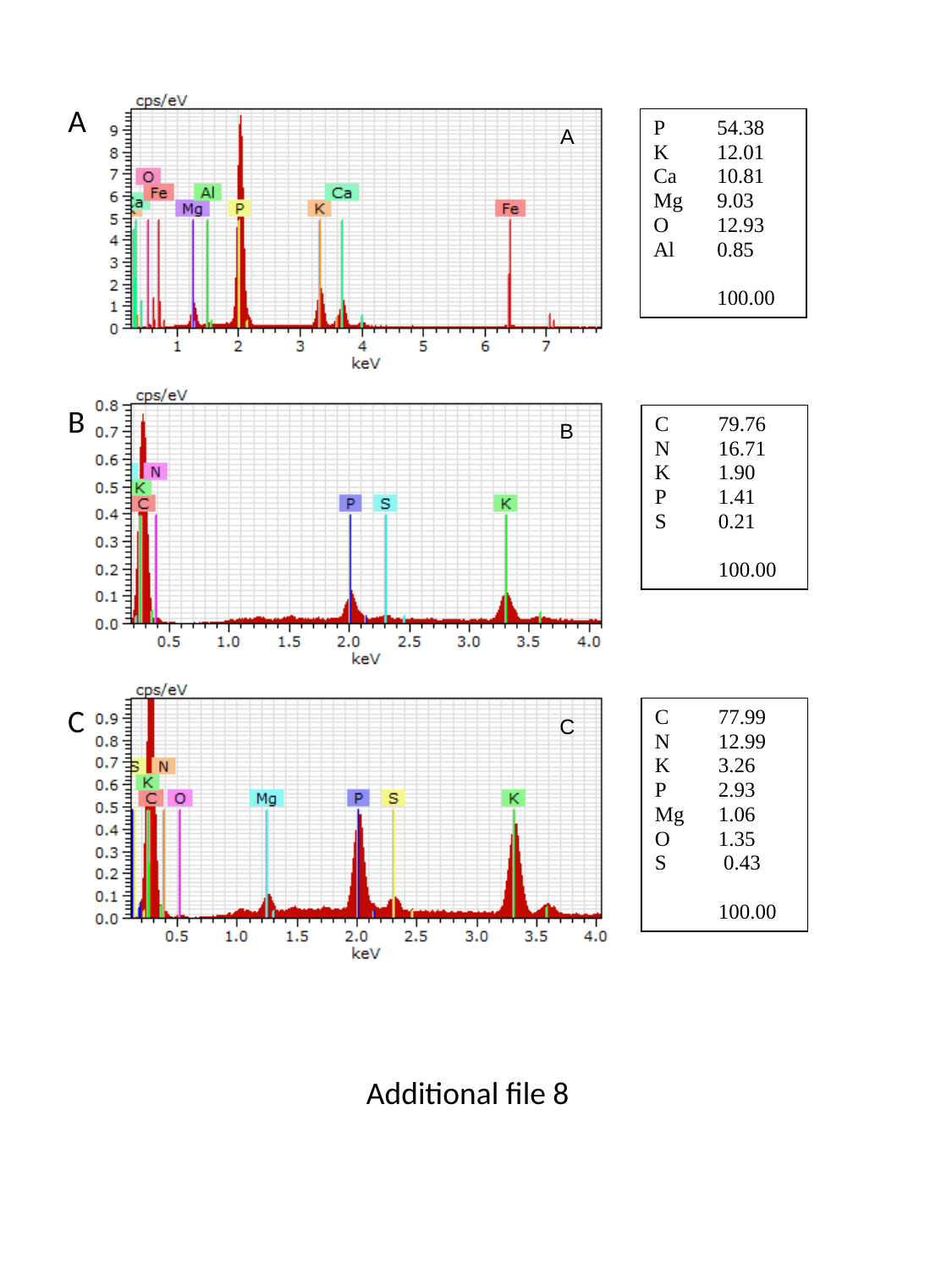

A
B
C
Additional file 8

Supplement: Additional file 8: — EDS spectra of the ash and bio-chars produced from biomass samples. (A) Ash from C. protothecoides; (B) bio-char from A. fumigatus/C. protothecoides; (C) bio-char from A. fumigatus. The distribution of elements is presented in atomic percent. [file 13068_2015_210_MOESM8_ESM.pptx]
