# Supplementary material for: Fungal-assisted algal flocculation: application in wastewater treatment and biofuel production
Source: Biotechnol Biofuels. 2015 Feb 15;8:24. doi: 10.1186/s13068-015-0210-6 (PMC4355497; doi:10.1186/s13068-015-0210-6)
Supplement: Additional file 2: — Table of fungal strains. [file 13068_2015_210_MOESM2_ESM.pptx]

## Slide 1
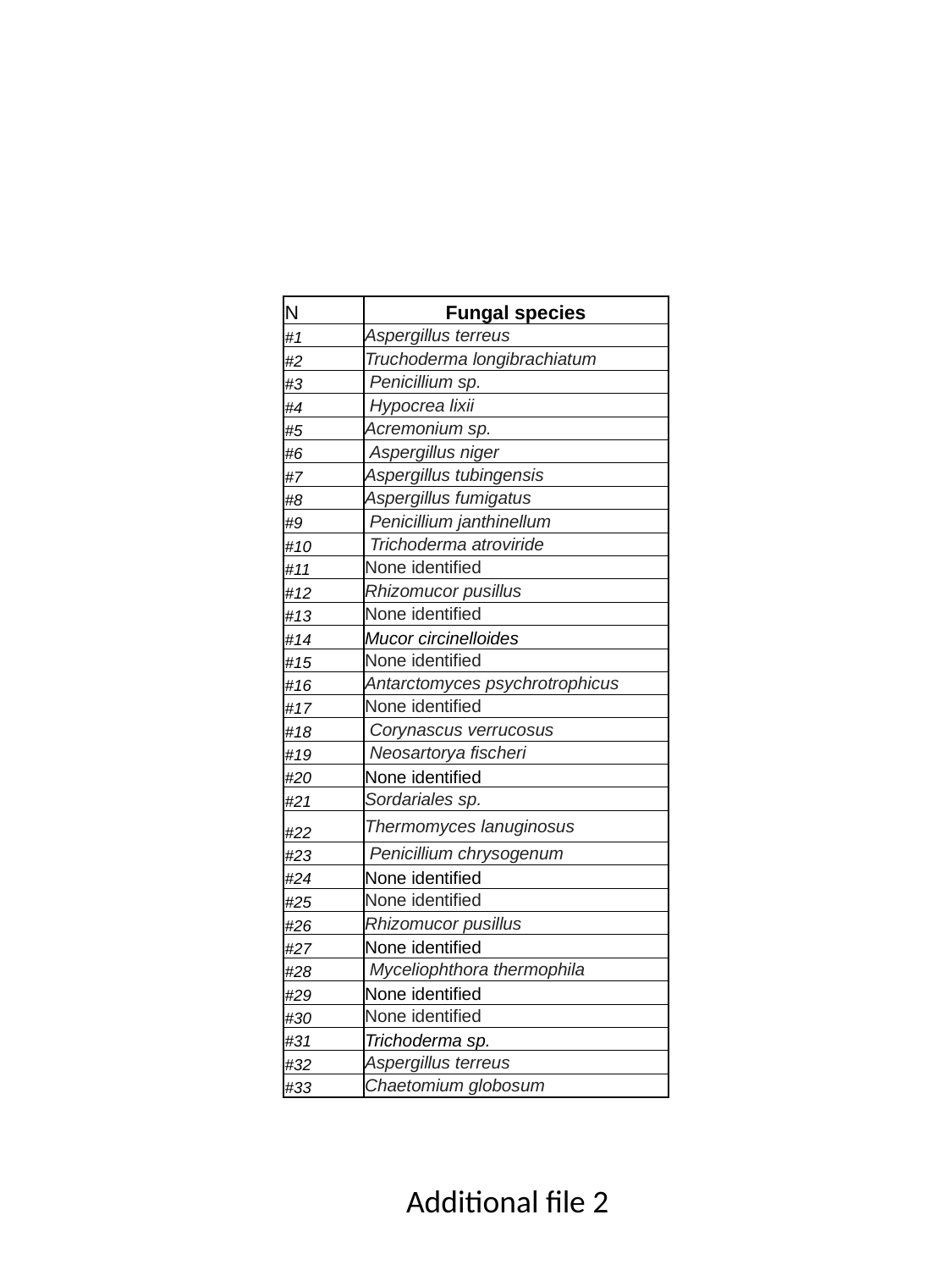

| N | Fungal species |
| --- | --- |
| #1 | Aspergillus terreus |
| #2 | Truchoderma longibrachiatum |
| #3 | Penicillium sp. |
| #4 | Hypocrea lixii |
| #5 | Acremonium sp. |
| #6 | Aspergillus niger |
| #7 | Aspergillus tubingensis |
| #8 | Aspergillus fumigatus |
| #9 | Penicillium janthinellum |
| #10 | Trichoderma atroviride |
| #11 | None identified |
| #12 | Rhizomucor pusillus |
| #13 | None identified |
| #14 | Mucor circinelloides |
| #15 | None identified |
| #16 | Antarctomyces psychrotrophicus |
| #17 | None identified |
| #18 | Corynascus verrucosus |
| #19 | Neosartorya fischeri |
| #20 | None identified |
| #21 | Sordariales sp. |
| #22 | Thermomyces lanuginosus |
| #23 | Penicillium chrysogenum |
| #24 | None identified |
| #25 | None identified |
| #26 | Rhizomucor pusillus |
| #27 | None identified |
| #28 | Myceliophthora thermophila |
| #29 | None identified |
| #30 | None identified |
| #31 | Trichoderma sp. |
| #32 | Aspergillus terreus |
| #33 | Chaetomium globosum |
Additional file 2
