# Supplementary material for: Fungal-assisted algal flocculation: application in wastewater treatment and biofuel production
Source: Biotechnol Biofuels. 2015 Feb 15;8:24. doi: 10.1186/s13068-015-0210-6 (PMC4355497; doi:10.1186/s13068-015-0210-6)
Supplement: Additional file 3: — Screening of fungal strains on carbon-free media for oil accumulation. (A) Phenotypic evaluation of fungal strains grown on carbon-free plates; 1) diameter of fungal growth area at time 0; 2) diameter of fungal growth area after 12 h; 3) diameter of fungal growth area after 24 h; (B) growth ratio of 33 fungal strains on carbon-free plates. [file 13068_2015_210_MOESM3_ESM.pptx]

## Slide 1
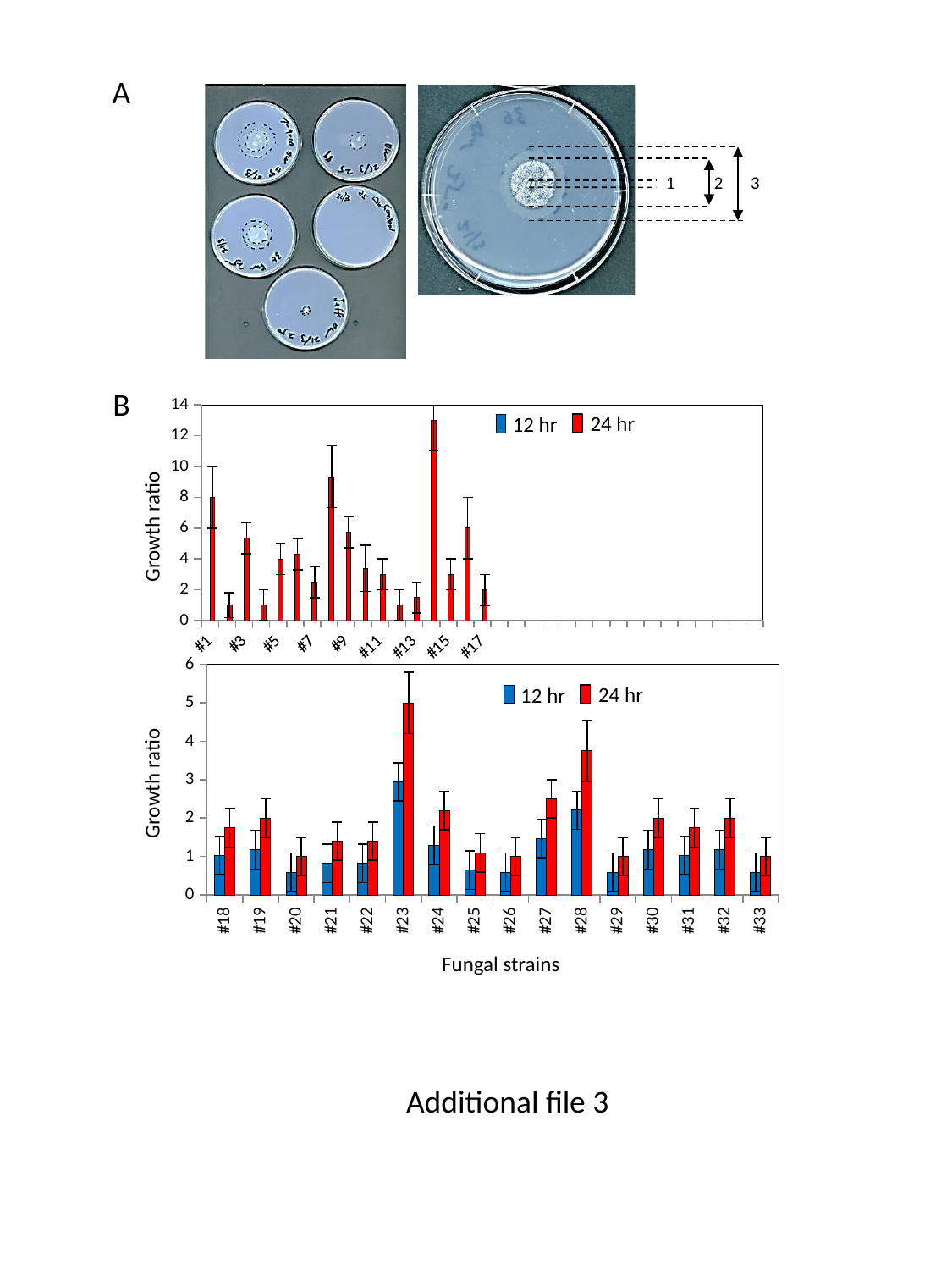

A
b
c
a
1
2
3
B
### Chart
| Category | | |
|---|---|---|
| #1 | 7.000000000000001 | 8.0 |
| #2 | 1.0 | 1.0 |
| #3 | 3.666666666666667 | 5.333333333333334 |
| #4 | 1.0 | 1.0 |
| #5 | 4.0 | 4.0 |
| #6 | 2.857142857142857 | 4.285714285714286 |
| #7 | 1.7499999999999998 | 2.5 |
| #8 | 6.666666666666667 | 9.333333333333334 |
| #9 | 2.2727272727272725 | 5.72727272727273 |
| #10 | 3.4 | 3.4 |
| #11 | 2.5 | 2.9999999999999996 |
| #12 | 1.0 | 1.0 |
| #13 | 1.4999999999999998 | 1.4999999999999998 |
| #14 | 6.0 | 13.0 |
| #15 | 2.0 | 3.0 |
| #16 | 4.333333333333334 | 6.0 |
| #17 | 1.1764705882352942 | 2.0 |24 hr
12 hr
Growth ratio
### Chart
| Category | | |
|---|---|---|
| #18 | 1.0294117647058825 | 1.75 |
| #19 | 1.1764705882352942 | 2.0 |
| #20 | 0.5882352941176471 | 1.0 |
| #21 | 0.8235294117647058 | 1.4 |
| #22 | 0.8235294117647058 | 1.4 |
| #23 | 2.9411764705882355 | 5.0 |
| #24 | 1.2941176470588236 | 2.2 |
| #25 | 0.6470588235294118 | 1.1 |
| #26 | 0.5882352941176471 | 1.0 |
| #27 | 1.4705882352941178 | 2.5 |
| #28 | 2.2058823529411766 | 3.75 |
| #29 | 0.5882352941176471 | 1.0 |
| #30 | 1.1764705882352942 | 2.0 |
| #31 | 1.0294117647058825 | 1.75 |
| #32 | 1.1764705882352942 | 2.0 |
| #33 | 0.5882352941176471 | 1.0 |24 hr
12 hr
Growth ratio
Fungal strains
Additional file 3
