# Supplementary material for: Fungal-assisted algal flocculation: application in wastewater treatment and biofuel production
Source: Biotechnol Biofuels. 2015 Feb 15;8:24. doi: 10.1186/s13068-015-0210-6 (PMC4355497; doi:10.1186/s13068-015-0210-6)
Supplement: Additional file 7: — Optical and SEM images of bio-char and inorganic ash. (A) Bio-char from A. fumigatus/C. protothecoides; (B) bio-char from A. fumigatus; (C) ash from C. protothecoides, (D) SEM of ash from C. protothecoides (magnification × 2,500). [file 13068_2015_210_MOESM7_ESM.pptx]

## Slide 1
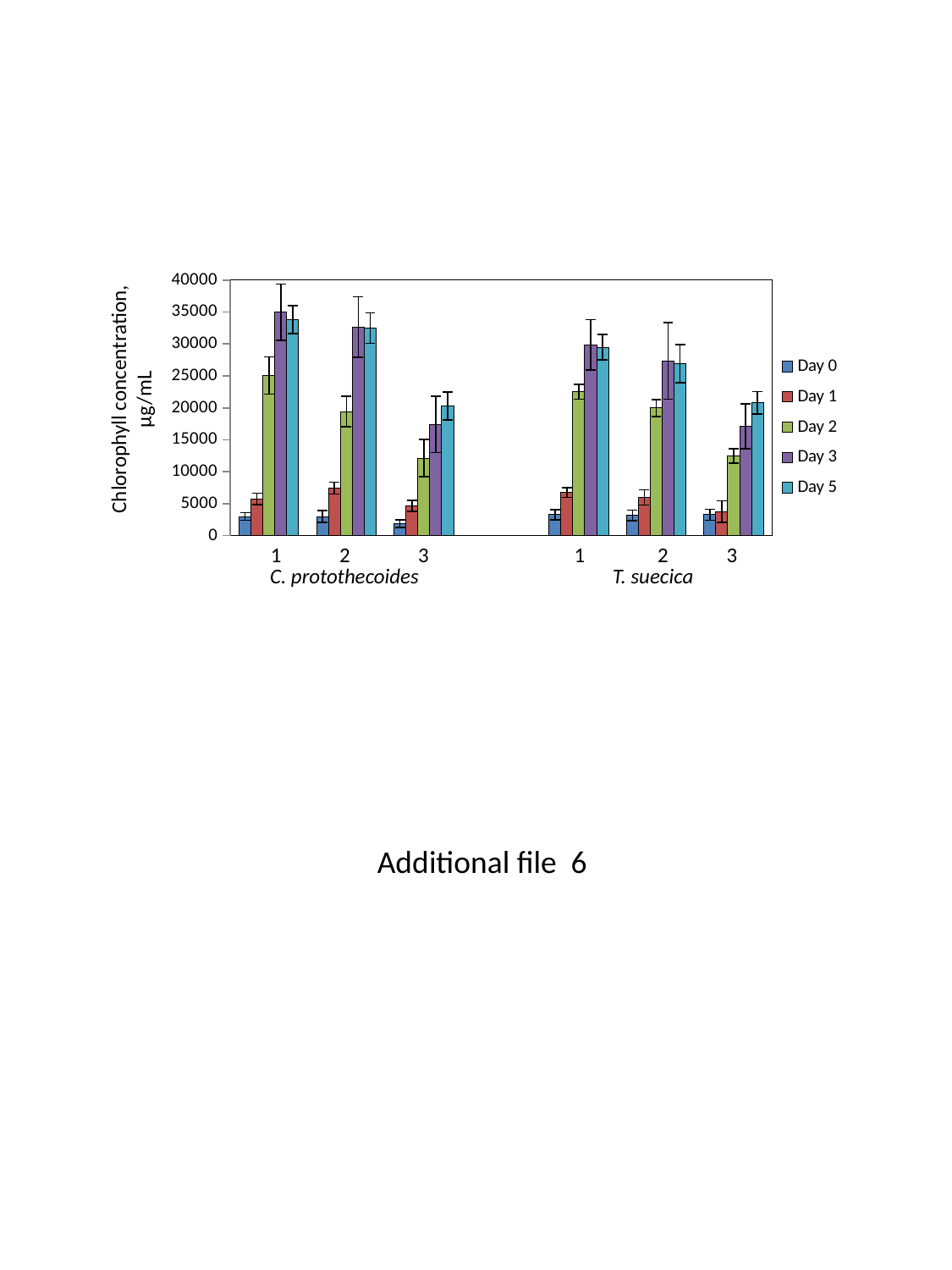

### Chart
| Category | Day 0 | Day 1 | Day 2 | Day 3 | Day 5 |
|---|---|---|---|---|---|
| C. protothecoides, control | 2997.2999999999997 | 5779.966666666666 | 25042.38 | 34958.53 | 33799.96666666667 |
| C. protothecoides, 5% TWS | 2997.2999999999997 | 7447.696666666666 | 19433.8 | 32673.6333333333 | 32470.49666666667 |
| C. protothecoides, 20% TWS | 1873.3124999999998 | 4654.81041666667 | 12146.124999999998 | 17421.0208333333 | 20294.060416666667 |
| | None | None | None | None | None |
| T. suecica, control
 | 3271.9666666666667 | 6737.966666666666 | 22524.46666666667 | 29864.966666666664 | 29496.633333333335 |
| T. suecica, 5% TWS
 | 3172.0909090909086 | 6010.272727272726 | 19972.545454545456 | 27355.424242424237 | 26922.39393939394 |
| T. suecica, 20% TWS
 | 3271.9666666666667 | 3756.4204545454536 | 12482.84090909091 | 17097.14015151515 | 20826.4962121212 |Chlorophyll concentration, μg/mL
1 2 3 1 2 3
C. protothecoides T. suecica
Additional file 6
